# Fracture zones in the Mid Atlantic Ridge lead to alterations in prokaryotic and viral parameters in deep-water masses

**DOI:** 10.3389/fmicb.2014.00264

**Published:** 2014-06-02

**Authors:** Simone Muck, Thomas Griessler, Nicole Köstner, Adam Klimiuk, Christian Winter, Gerhard J. Herndl

**Affiliations:** ^1^Department of Limnology and Oceanography, Center of Ecology, University of ViennaVienna, Austria; ^2^Department of Biological Oceanography, Royal Netherlands Institute for Sea Research (NIOZ)Den Burg, Netherlands

**Keywords:** deep sea, microbial communities, mixing zones, North Atlantic, prokaryotes, Vema Fracture Zone, viruses

## Abstract

We hypothesized that mixing zones of deep-water masses act as ecotones leading to alterations in microbial diversity and activity due to changes in the biogeochemical characteristics of these boundary systems. We determined the changes in prokaryotic and viral abundance and production in the Vema Fracture Zone (VFZ) of the subtropical North Atlantic Ocean, where North Atlantic Deep Water (NADW) and Antarctic Bottom Water (AABW) are funneled through this narrow canyon and therefore, are subjected to intense vertical mixing. Consequently, salinity, potential temperature, oxygen, PO_4_, SiO_4_, NO_3_ were altered in the NADW inside the VFZ as compared to the NADW outside of the VFZ. Also, viral abundance, lytic viral production (VP) and the virus-to-prokaryote ratio (VPR) were elevated in the NADW in the VFZ as compared to the NADW outside the VFZ. In contrast to lytic VP, lysogenic VP and both the frequency of lytically (FIC) and lysogenically infected cells (FLC) did not significantly differ between in- and outside the VFZ. Generally, FIC was higher than FLC throughout the water column. Prokaryotic (determined by T-RFLP) and viral (determined by RAPD-PCR) community composition was depth-stratified inside and outside the VFZ. The viral community was more modified both with depth and over distance inside the VFZ as compared to the northern section and to the prokaryotic communities. However, no clusters of prokaryotic and viral communities characteristic for the VFZ were identified. Based on our observations, we conclude that turbulent mixing of the deep water masses impacts not only the physico-chemical parameters of the mixing zone but also the interaction between viruses and prokaryotes due to a stimulation of the overall activity. However, only minor effects of deep water mixing were observed on the community composition of the dominant prokaryotes and viruses.

## Introduction

In the marine environment, frontal systems (vertical boundary systems of water masses) are generally considered hotspots of diversity and activity (Doney et al., 2004; Longhurst, 2007; Ribalet et al., 2010), hence show typical characteristics of ecotones. Extending the ecotone concept to deep-waters, we hypothesized that the mixing zones of deep-water masses can also be seen as ecotones and consequently, might also be sites of higher prokaryotic diversity and productivity and linked to that, of higher viral activity and diversity than the adjacent parent water masses.

In the deep ocean, mixing of water masses is particularly pronounced in areas where different water masses are funneled through canyons such as in the fracture zones of the Mid Atlantic Ridge. These fracture zones represent major conduits of deep waters between the eastern and the western basin of the Atlantic and thus, represent ideal sites to study the effect of mixing of deep-water masses on microbial and viral activity and diversity. Also, water masses funneled through fracture zones are potentially subjected to nutrient and material input from the slopes and bottom due to resuspension of sediments (Sloth et al., 1996). Hence, these fracture zones might be sites of larger changes in the microbial and viral community in specific water masses than in the same water mass outside the fracture zones. In this study, we focused on the Vema Fracture Zone (VFZ) of the Mid-Atlantic Ridge (Demidov et al., 2007), a well-studied region with intense mixing of well-defined deep-water masses and topography (Eittreim et al., 1983; Fischer et al., 1996), to study the interaction and changes in parameters describing viral and prokaryotic communities.

Viruses are generally considered to be highly host-specific parasites (Ackermann and DuBow, 1987) playing an important ecological role in the marine environment by controlling the abundance and diversity of the microbial community either indirectly or directly as described by the “killing the winner” model (Thingstad and Lignell, 1997). Several studies point out, however, that not all marine viruses are host-specific and some might have a broad host range (Rohwer, 2003; Sano et al., 2004; Holmfeldt et al., 2007). In the open ocean, viral and prokaryotic abundance are positively correlated (Fuhrman, 1999; Magagnini et al., 2007). Both, viral and prokaryotic abundance significantly decrease with depth in the global ocean (Aristegui et al., 2009). However, viral abundance declines less with depth than prokaryotic abundance, resulting in an increase of the virus-to-prokaryote ratio (VPR) from about 10 in the surface waters to 30–100 in the bathypelagic waters of the Atlantic (Parada et al., 2007; De Corte et al., 2010). This increase in the VPR with depth cannot be related to the predominating lysogenic life strategy of viruses in the bathypelagic waters, thought to be an adaption to low host abundances (Weinbauer et al., 2003). Potentially, the lysogenic viral cycle might be induced as a stress response due to organic carbon limitation commonly reported for the deep ocean (Wilson and Mann, 1997). Estimating the rate at which viruses are produced is key to understand the impact of viruses on microbial communities and their role in the biogeochemical cycling of organic matter (Wommack and Colwell, 2000; Wilhelm et al., 2002; Mei and Danovaro, 2004). Viral production is positively correlated to prokaryotic respiration and growth rates (Glud and Middelboe, 2004; Mei and Danovaro, 2004; Weinbauer, 2004; Middelboe et al., 2006; Danovaro et al., 2008, 2011). Hence, changes in the growth rates of deep-water prokaryotic communities due to turbulent mixing might leave an imprint in viruses as well.

To determine the successional changes in the viral and prokaryotic community in the deep water masses during their flow through narrow fracture zones, we determined selected abiotic and biotic parameters throughout the water column along a transect through the VFZ and compared them with the parameters obtained in the same water masses outside the VFZ. Specifically, we determined the relation between the abundance and production of viruses and prokaryotes and analyzed possible changes in the respective community composition. We expected that several microbial parameters are elevated in the deep water masses of the VFZ as compared to these water masses outside the VFZ leading ultimately to alterations in the prokaryotic and viral community.

## Materials and methods

### Study area and sampling

The VFZ of the Mid-Atlantic Ridge is, besides the Romanche Fracture Zone, the main conduit of the Antarctic Bottom Water (AABW) entering the eastern basin of the North Atlantic (Tomczak and Godfrey, 1994). The VFZ is located along 11°N from 45 to 40°W with a width of 8–20 km and a maximum depth of approximately 5200 m (Tomczak and Godfrey, 1994). AABW and North Atlantic Deep Water (NADW) are flowing through the VFZ from west to east with an average velocity of 30 cm s^−1^ (Demidov et al., 2007), transporting approximately 3.5 Sv (1 Sv = 10^6^ m^3^ s^−1^) of water with strong mixing caused by vertical and lateral friction. AABW is found below 4000 m depth and characterized by low temperature (0–2°C), low salinity and high nutrient content, particularly in silicate (Demidov et al., 2007). The oxygen-rich NADW is overlying the AABW and originates in the Labrador Sea and the Greenland-Iceland-Scotland overflow region and is characterized by higher temperature and salinity as well as lower nutrient concentrations than the AABW (Tomczak and Godfrey, 2001). The Antarctic Intermediate Water (AAIW), originating in the Antarctic Convergence Zone, is flowing through the VFZ in the opposite direction as the AABW and the NADW at a depth of around 900–1500 m, transporting nitrate-rich water from the eastern Atlantic basin through the VFZ into the western basin (McCartney et al., 1991). Hence, there is intensive mixing between the AAIW and the upper NADW and between the lower NADW and the AABW in the VFZ. More information on the current pattern of the major water masses of the North Atlantic and the water column structure of the VFZ is given elsewhere (Tomczak and Godfrey, 1994; Morozov et al., 2010).

**Figure 1:**
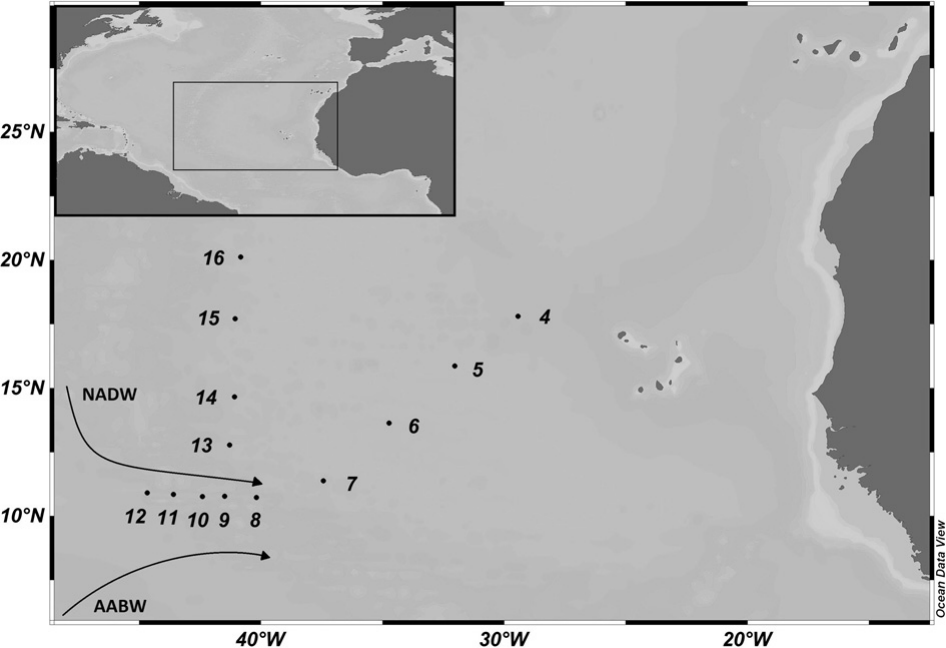
**Map of the study area in the (sub)tropical North Atlantic Ocean with the sampling sites occupied during the MOCA cruise in 2010**. The cruise track is divided into northern (Stations 13–16) and eastern (Stations 4–7) section and the Vema Fracture Zone (Stations 8–12). The flow direction of the main deep-water masses, the North Atlantic Deep Water (NADW) and Antarctic Bottom Water (AABW) is indicated.

Sampling was carried out in the (sub) tropical North Atlantic Ocean during the MOCA cruise on board R/V *Pelagia* in October 2010 (Figure 1). Samples were collected at 13 stations at six distinct water layers: at 100 m depth corresponding to the lower end of the euphotic layer, the oxygen minimum zone (OMZ), the AAIW, the Upper and Lower North Atlantic Deep Water (UNADW, LNADW) and the AABW. Water masses were identified based on their salinity and temperature characteristics during the downcast of the CTD (conductivity-temperature-depth) rosette sampler equipped with 18 25-L Niskin bottles. Based on the water mass transport through the VFZ, the samples collected along this transect were divided into three sections for subsequent analyses: water masses in the VFZ and the regions north and east of the VFZ coined thereafter, northern and eastern section.

### Inorganic nutrients

A TRAACS autoanalyzer was used for spectrophotometric determination of the dissolved inorganic nutrients (SiO_4_, PO_4_, NO_2_, and NO_3_) following the Joint Global Ocean Fluxes Study recommendations (Gordon et al., 1993). Samples were filtered through 0.2 μm Acrodisc filters and analyzed immediately. SiO_4_ was measured as a blue reduced silicon-molybdenum complex at a wavelength of 880 nm. Ascorbic acid was used as reductant and oxalic acid for preventing interference of phosphate. PO_4_ was detected by formation of a molybdenum-blue complex at 880 nm. NO_2_ was determined after diazotation with sulfanilamide and *N*-(1-naphtyl)-ethylene diammonium dichloride forming a reddish-purple dye complex at 540 nm wavelength. NO_3_ was reduced in a copper-cadmium coil to nitrite using imidazole as a buffer and then measured as nitrite.

### Prokaryotic and viral abundance

Prokaryotic and viral abundance were determined by flow cytometry (FCM) (Marie et al., 1999). Samples were fixed with glutaraldehyde (0.5% final concentration), flash-frozen in liquid nitrogen and stored at −80°C until analysis. Samples were enumerated on a FACSAria II Cell sorter (Becton Dickinson) as previously described (Marie et al., 1999; Brussaard, 2004). Based on plots of side scatter vs. green fluorescence, three different prokaryotic populations (high nucleic acid and high side scatter—HNA-HS, high nucleic acid and low side scatter—HNA-LS, low nucleic acid—LNA) and three different viral populations (high fluorescence—VirHigh, medium fluorescence—VirMed and low fluorescence—VirLow) were distinguished (Brussaard et al., 2010).

### Prokaryotic community composition

For the analysis of the bacterial (BCC) and archaeal community composition (ACC), 20 L of seawater were concentrated using a Pellicon tangential-flow ultrafiltration device with a 0.22 μm pore-size cartridge. The concentrate was filtered onto a 0.22 μm pore-size membrane filter (GVWP) and stored in cryovials at −80°C after flash-freezing in liquid nitrogen. DNA extraction was performed using an UltraClean Soil DNA Isolation Kit (MoBio Laboratories) applying the protocol of the manufacturer. DNA extracts were stored at −80°C. PCR and terminal-restriction fragment length polymorphism (T-RFLP) were used for the characterization of the prokaryotic community (Moeseneder et al., 2001a,b). Briefly, 1 μL of DNA extract was used as template in a 50 μL PCR mixture. To amplify the 16S rRNA genes, different primer pairs were used for Bacteria and Archaea: the Bacteria-specific 27F-FAM (Moeseneder et al., 1999) in combination with 1492R-VIC (Lane, 1991) and the Archaea-specific 21F-FAM (Moeseneder et al., 2001a,b) combined with the 958R-VIC (DeLong, 1992) primer (Thermo Scientific). Both primers (forward and reverse) were 5′ end-labeled with the two fluorescent reporter dyes FAM (phosphoramidite fluorochrome 5-carboxy-fluorescein) and VIC (6-carboxy-4′, 5′- dichloro-2′, 7′-dimethoxyfluorescein) to obtain a blue and green signal in the sequencer analysis, respectively. Samples were amplified by an initial denaturation step at 95°C for 5 min, followed by 30 cycles of denaturation at 95°C for 1 min, annealing at 55°C for 1 min and elongation at 72°C for 1 min. The final elongation step was performed at 71°C for 30 min (Janse et al., 2004). A negative control (autoclaved and UV-treated MilliQ water instead of template DNA) was included in each PCR run to check for potential contamination. PCR products were checked by gel electrophoresis for successful PCR, the 1.0% agarose gel was stained with a working solution of SYBR Gold. The PCR products were purified using the PCR Extract Mini Kit (5 Prime) following the manufacturer's protocol and quantified with a Nanodrop spectrophotometer. The enzymatic digest of the purified PCR products (5–10 μL) was performed at 37°C for 12 h followed by incubation at 65°C for 20 min to inactivate the tetrameric restriction enzyme *Hha*I. For further analysis on an ABI 3130XL automated sequencer (Applied Biosystems), 1 μL of restriction digest was added to 10 μL Hi-Di formamide (highly deionized formamide for capillary electrophoresis) (Applied Biosystems) and 0.25 μL of 1200 LIZ standard (GeneScan), heated to 95°C for 3 min and immediately put on ice before analysis. The size of the fluorescently labeled fragments was determined by comparison with the internal standard. The output was analyzed with the software Peak Scanner version 1.0 (Applied Biosystems). The threshold of peaks was set to 25 for the blue and green channel and 20 for the size standard (orange) to distinguish signal from noise. Thus, peaks were defined as operational taxonomic units (OTUs), with a resolution of ±1 basepair (bp) for short fragments and ±4 bp for fragments >1000 bp. Fragments with a size of 25–1200 bp according to the size standard were used for further analyses.

### Viral community composition

After prokaryotes were removed from the seawater for determining the prokaryotic community composition as described above, the 0.2 μm filtered water was concentrated using spiral-wound ultrafiltration cartridges (Amicon) and a Vivaflow 200 ultrafiltration device (both 100 kDa cut-off). The obtained viral concentrate (30–50 mL) was flash-frozen in liquid nitrogen and stored at −80°C aboard the ship.

In the lab, the samples were brought up to room temperature and filtered through a 0.2 μm Acrodisc Syringe Filter to ensure removal of prokaryotic cells. Subsequently, the samples were concentrated to a final volume of approximately 200 μL using the Amicon Ultra-15 Centrifugal Filter Units with Ultracell-100 membranes (Millipore), following the protocol of the manufacturer. DNA extraction was performed with a QIAmp MiniElute Virus Spin Kit (Qiagen) according to the manufacturer's protocol. For the randomly amplified polymorphic DNA (RAPD)-PCR, the primers CRA-22 and OPA-13 (Wommack et al., 1999) were used. Only one primer was used in each reaction, acting as both forward and reverse primer. The total volume of one PCR reaction mixture was 50 μL. A negative control was used for each run to check for potential contamination. Samples were amplified according to the following protocol: initial denaturation and enzyme activation at 94°C for 10 min, followed by 30 cycles of denaturation at 94°C for 30 sec, annealing at 35°C for 3 min and elongation at 72°C for 1 min. The cycle was completed by final elongation at 72°C for 30 min. The PCR products were separated by gel electrophoresis on 2.5% agarose gels. The gel was stained with a working solution of SYBR Gold. Based on the DNA rulers SmartLadder (200–10000 bp) and the Fermentas Gene Ruler (250–10000 bp), the obtained bands were sized using the Quantity One Software.

### Prokaryotic heterotrophic production

^3^H-leucine incorporation (specific activity: 595.7 × 10^10^ Bq mmol^−1^; final concentration 5–10 nmol L^−1^) into prokaryotic cells was used to estimate prokaryotic heterotrophic production (PHP) (Simon and Azam, 1989). Ten-40 mL of unfiltered seawater and blanks were incubated in triplicate in the dark. The blanks were fixed with concentrated formaldehyde (4% final concentration, v/v; 0.2 μm filtered) 10 min prior to the addition of the tracer. Subsequently, the samples and blanks were incubated at in situ temperature for 4–48 h (depending on the expected activity). Thereafter, the live samples were fixed with formaldehyde (4% final conc.) and filtered onto 0.2 μm polycarbonate filters (Millipore; 25 mm diameter) using Millipore HAWP supporting filters. The filters were rinsed twice with 5 mL ice-cold 5% trichloroacetic acid for 5 min and then transferred into 20 mL scintillation vials. After drying the filters, 8 mL of scintillation cocktail (Filter Count, Perkin-Elmer) was added. After 18 h, the radioactivity was counted in a liquid scintillation counter (Perkin Elmer, TriCarb 2910TR). Subsequently, the amount of leucine incorporated into prokaryotic biomass was calculated.

### Viral production

The virus dilution approach (Wilhelm et al., 2002) was used to estimate the frequency of infected cells (FIC), the frequency of lysogenic cells (FLC) and the lytic viral production (VP). Per station, these viral parameters were determined in three water masses (OMZ, LNADW, AABW). For each experiment, the prokaryotic community was concentrated from an original total volume of 21 L using Pellicon filter-cassettes (0.22 μm) in combination with a Vivaflow 200 ultrafiltration (0.22 μm pore-size; Vivasciences). To generate virus-free seawater, the 0.22 μm filtrate was passed through a Vivaflow 200 ultrafiltration device with a molecular weight cut-off of 100 kDa. Ten mL of prokaryotic concentrate was added to 40 mL of the corresponding virus-free ultrafiltrate and incubated at in situ temperature in the dark after addition of mitomycin C.

The experiments were set up at 11 stations. Per depth, one set of experiments contained a final concentration of 1 μg mL^−1^ mitomycin C (Sigma) while the other set contained no mitomycin C. Mytomycin C is a DNA damaging agent inducing a prokaryotic stress response and therefore, leads to the induction of the lytic cycle of lysogenic viruses (Weinbauer and Suttle, 1996). Subsamples (2 mL) for determining viral and prokaryotic abundance were taken every 4 h for a total of approximately 36 h, fixed with glutaraldehyde, flash-frozen in liquid nitrogen and stored at −80°C until analysis. Flow cytometry was used for enumerating prokaryotes and viruses as described above. FIC, FLC, and VP were calculated according to the following formulas:

Frequency of infected cells (Winter et al., 2004a):

(1) $$FIC(\%)=\;\left[\frac{\left(V_{\max1}-V_{\min1}\right)+\left(V_{\max N}-V_{\min N}\right)}{burst\;size\;\times \;P_{0}}\right]\;\times \;100$$

Frequency of lysogenic cells (Weinbauer et al., 2003):

(2) $$FLC(\%)=\;\left[\frac{\begin{array}{l} \left(V_{\max1(+MitC)}-V_{control1}\right)+(V_{\max2(+MitC)}\\ \;-V_{control2})+\left(V_{\max N(+MitC)}-V_{controlN}\right) \end{array}}{burst\;size\;\times \;P_{0}}\right]\;\times \;100$$

Lytic VP (Winter et al., 2004a):

(3) $$Lytic\;VP=\;\frac{\left(V_{\max1}-V_{\min1}\right)+\left(V_{\max2}-V_{\min2}\right)+\left(V_{\max N}-V_{\min N}\right)}{t_{\max N}-t_{\min1}}$$

Lysogenic VP (Weinbauer et al., 2003):

(4) Lysogenic VP =  FLC – FIC

In equation (1), FIC is the frequency of infected cells as percentage of total prokaryotic abundance, *V*_max_—the highest viral abundance, *V*_min_—lowest viral abundance and *P*_0_—initial prokaryotic abundance before the addition of mitomycin C. The burst size (number of viruses released per cell due to viral lysis) was assumed to be 30 (Parada et al., 2006). If multiple peaks in viral abundance were detected during the time course of the experiment, FIC was calculated separately for each peak and the values added (Winter et al., 2004a). FLC (Equation 2) was defined as the difference of viral abundance between mitomycin C treated samples [*V*_(+*MitC*)_] and control incubations (*V*_*control*_) (Weinbauer et al., 2003). Lytic VP (Equation 3) was calculated as the slope between each minimum (*V*_min_) and the following maximum (*V*_max_) viral abundance divided by the incubation time in hours (*t*_max_ − t_min_). Subscripts 1, 2 and n denote the peaks 1, 2, and n, respectively. In equation (4), the lysogenic VP was calculated as the difference between VP obtained in mitomycin C treated samples (FLC) and samples without mitomycin C added (FIC).

### Statistical analysis

Spearman's rank correlations were performed to analyze correlations between all pairs of measured parameters. Only statistically significant (*p* ≤ 0.05) and relevant (−0.5 > *r*_*s*_ > 0.5) correlation coefficients were considered for further interpretation of the results. To evaluate differences between the water masses of the different sections, the One-Way analysis of variance (ANOVA) with a *post-hoc* Bonferroni correction was performed. When normal distribution (Shapiro-Wilk test) was not obtained, Kruskal-Wallis ANOVA on rank was performed and, when significant differences (*p* < 0.05) were observed, a *post-hoc* Dunn's test was done. Results of the statistical analyses are given in the respective tables. Normal distribution of abundance and correlation analyses were performed using the SPSS software and, ANOVA and ANOVA on rank were performed with SigmaPlot 12.0 (Systat Software, Chicago, IL, USA). SPSS Statistics Software 2.0 was used to perform an analysis of covariance (ANCOVA) to test the relation of the regression slopes between the similarity in community composition and the distance between samples inside and north of the VFZ. Absence and presence of OTUs of individual samples determined by T-RFLP and RAPD-PCR were further analyzed using the Primer-E software to determine the Jaccard similarity, applying the group average method for establishing dendrograms.

## Results

### Oceanographic conditions

The temperature and salinity characteristics as well as the oxygen concentrations of the main water masses encountered along the transect through the VFZ and the northern and eastern section are given in Table S1. In the deep-water masses of the VFZ, salinity was significantly lower than in the northern section and the eastern section. Also, the potential temperature of the AABW, the AAIW and the LNADW was significantly lower in the VFZ than outside the VFZ. The UNADW at approximately 2000 m depth exhibited a significantly higher oxygen concentration in the VFZ than outside the VFZ (Table S1).

The inorganic nutrient concentrations of the water masses encountered in the VFZ are shown in Table S2. In the VFZ, PO_4_, NO_3_, and SiO_4_ concentrations were significantly higher in the AABW and significantly lower in the UNADW than in the corresponding water masses outside the VFZ. In the LNADW, only SiO_4_ concentrations were significantly higher in the VFZ than in the northern section (Table S2).

Collectively, the physical and chemical parameters of the individual water masses in- vs. outside of the VFZ indicate that the deep-water masses of the VFZ were subjected to mixing with the adjacent water mass.

### Distribution of prokaryotic and viral communities

Generally, total prokaryotic and viral abundance decreased with depth by one order of magnitude from the 100 m depth horizon to the UNADW (Table 1). In the VFZ, prokaryotic abundance was significantly lower at 100 m depth than at the same depth in the northern section. Viral abundance was significantly higher in the UNADW of the VFZ than in the UNADW of the eastern section. At all stations, prokaryotic abundance was highly correlated to viral abundance (Table S3) and to PHP (Table 2).

**Table 1:** **Prokaryotic and viral parameters determined by flow cytometry during the MOCA cruise sampled in six different water masses north (Northern section), within and east (Eastern section) of the Vema Fracture Zone in the (sub)tropical North Atlantic Ocean**.

**Water mass**	**PA (*N* × 10^5^ cells mL^−1^)**	**VA (*N* × 10^5^ counts mL^−1^)**	**VPR**	**PHP (nmol leu m^−3^ d^−1^)**	**HNA-LS (%)**	**HNA-HS (%)**	**LNA (%)**	**VirHigh (%)**	**VirMed (%)**	**VirLow (%)**	***N***
**NORTHERN SECTION**											
100 m	**4.07** (0.86)	87.55 (1.13)	21.9 (2.4)	**260.80** (76.32)	32.1 (9.5)	18.1 (5.7)	49.8 (7.3)	10.9 (0.9)	48.6 (5.0)	40.4 (5.8)	4
OMZ	0.58 (0.18)	9.63 (0.22)	16.8 (1.4)	2.89 (1.26)	37.6 (3.1)	8.0 (0.5)	54.4 (3.4)	4.5 (0.7)	51.6 (2.8)	43.9 (3.4)	4
AAIW	0.28 (0.05)	5.55 (0.04)	20.3 (2.8)	**0.30** (0.06)	37.3 (1.7)	10.2 (1.1)	52.2 (1.9)	4.1 (1.1)	48.5 (3.6)	47.3 (4.7)	4
UNADW	0.13 (0.03)	4.58 (0.07)	35.7 (2.6)	0.12 (0.06)	36.6 (1.6)	**18.2** (1.8)	45.2 (0.7)	4.2 (0.4)	46.6 (3.4)	49.2 (3.8)	4
LNADW	0.11 (0.01)	4.19 (0.05)	38.2 (3.6)	0.05 (0.04)	34.2 (1.9)	21.5 (1.3)	44.2 (1.4)	4.1 (0.5)	44.5 (2.1)	51.5 (2.4)	4
AABW	0.13 (0.01)	4.71 (0.05)	37.2 (1.8)	0.05 (0.03)	34.8 (1.7)	20.8 (1.8)	44.4 (1.2)	4.7 (0.7)	44.1 (2.1)	51.2 (2.7)	4
**VEMA FRACTURE ZONE**											
100 m	**2.60** (0.58)	57.60 (0.56)	22.6 (3.7)	**124.44** (20.34)	33.1 (1.4)	16.1 (1.6)	50.8 (1.5)	8.7 (0.7)	52.0 (8.3)	39.3 (8.8)	3
OMZ	0.80 (0.14)	13.02 (0.24)	16.7 (3.9)	3.10 (0.69)	39.2 (2.4)	7.9 (0.9)	52.9 (2.9)	**5.1** (1.5)	56.2 (7.6)	38.7 (9.1)	5
AAIW	0.38 (0.07)	7.17 (0.20)	18.4 (2.0)	**0.50** (0.04)	40.1 (3.3)	9.5 (0.9)	50.4 (4.1)	**4.8** (0.8)	45.8 (3.8)	49.4 (3.3)	3
UNADW	0.18 (0.01)	6.43* (0.06)	35.8* (5.3)	0.12 (0.02)	37.8 (1.6)	**15.5** (0.8)	46.8 (0.8)	5.2 (0.8)	57.3* (8.5)	37.5* (9.0)	3
LNADW	0.12 (0.01)	4.20 (0.04)	35.1 (3.4)	0.07 (0.01)	35.7 (1.3)	19.2 (1.7)	45.1 (2.8)	**4.0** (0.8)	44.5 (8.8)	51.4 (9.5)	5
AABW	0.15 (0.01)	5.64 (0.08)	36.8 (6.0)	0.11 (0.01)	37.2 (1.0)	17.7 (0.4)	44.1 (0.6)	5.8 (1.6)	53.0 (10.3)	41.2 (11.8)	3
**EASTERN SECTION**											
100 m	1.45 (0.27)	38.84 (1.88)	25.8 (7.3)	90.32 (35.28)	30.1 (6.2)	14.3 (2.7)	55.6 (3.5)	13.2 (2.8)	52.6 (16.1)	34.2 (16.4)	4
OMZ	0.82 (0.28)	10.48 (0.36)	13.1 (4.2)	3.74 (1.64)	37.3 (6.2)	8.2 (0.8)	54.4 (5.5)	**10.7** (2.3)	44.6 (25.3)	44.7 (24.8)	4
AAIW	0.33 (0.02)	4.61 (0.14)	13.9 (4.3)	0.37 (0.08)	36.2 (1.3)	10.8 (1.2)	53.0 (0.4)	**10.2** (1.1)	40.6 (17.6)	49.2 (18.2)	4
UNADW	0.17 (0.02)	2.28* (0.10)	13.0* (4.8)	0.06 (0.02)	36.6 (1.9)	15.7 (0.3)	47.7 (1.6)	7.1 (4.3)	13.5* (7.2)	79.4* (11.4)	4
LNADW	0.12 (0.01)	4.69 (0.04)	38.2 (3.2)	0.04 (0.01)	37.7 (1.4)	16.9 (0.8)	45.4 (1.9)	**8.5** (0.5)	71.3 (0.5)	20.2 (0.8)	4
AABW	0.13 (0.01)	5.04 (0.04)	40.7 (6.1)	0.04 (0.01)	37.1 (1.9)	19.6 (1.2)	43.4 (2.6)	9.7 (0.7)	56.8 (18.4)	33.5 (18.2)	4

Numbers in bold without and with an asterisk show significant differences (p ≤ 0.05 or p ≤ 0.01, respectively) of individual parameters between the Vema Fracture Zone and the northern and eastern stations. Abbreviations: 100 m, bottom of euphotic layer; HNA-HS, percentage of high nucleic acid prokaryotes with high scatter; HNA-LS, percentage of high nucleic acid prokaryotes with low scatter; LNA, percentage of low nucleic acid prokaryotes; N, number of samples; PA, prokaryotic abundance; PHP, prokaryotic heterotrophic production measured by leucine incorporation rate; VA, viral abundance; VirHigh, percentage of high fluorescent viruses; VirLow, percentage of low fluorescent viruses; VirMed, percentage of medium fluorescent viruses; VPR, virus-to-prokaryotes ratio; Values are average values for all parameters except VPR. Standard deviation is given in brackets.

**Table 2:** **Spearman's rank correlation coefficients determining the relationship of prokaryotic and viral populations to the physico-chemical parameters measured, prokaryotic and viral production and frequency of infected cells within the three sections (Northern section, Vema Fracture Zone, eastern section)**.

	**Depth**	**Salinity**	**Pot. Temp**.	**Oxygen**	**SiO_4_**	**PO_4_**	**NO_2_**	**NO_3_**	**VPR**	**PHP**	**Lysogenic VP**	**FIC**	**FLC**
**NORTHERN SECTION**													
Prok. abundance	−0.844	0.553	0.831	−0.619	−0.823	–	–	–	−0.789	0.877	–	–	–
HNA-HS	0.574	–	−0.545	0.744	0.531	−0.532	–	−0.530	0.771	−0.570	–	–	**0.720**
HNA-LS	–	–	–	–	–	–	–	–	–	–	–	**−0.626**	–
LNA	−0.585	–	0.584	−0.791	−0.590	–	–	–	−0.639	0.643	–	–	**−0.678**
Viral abundance	−0.830	0.601	0.821	−0.579	−0.822	–	–	–	−0.693	0.863	–	–	–
VirHigh	–	–	–	–	–	–	–	–	–	–	–	–	–
VirMed	−0.598	–	0.521	−0.538	**−0.501**	–	–	–	−0.689	0.554	–	–	**−0.762**
VirLow	0.700	–	−0.622	–	0.602	–	–	–	0.657	−0.695	–	–	–
PHP	−0.932	0.676	0.925	−0.751	−0.910	–	–	–	−0.742	–	–	–	–
VPR	0.783	–	−0.764	0.813	0.745	–	–	–	–	−0.742	–	**0.606**	–
FLC	0.820	−0.870	−0.820	**0.770**	0.820	–	–	–	–	–	–	–	–
**VEMA FRACTURE ZONE**													
Prok. abundance	−0.888	–	0.896	−0.743	−0.863	–	0.550	–	−0.725	0.960	–	–	–
HNA-HS	−0.678	–	−0.685	0.656	0.624	–	–	**−0.537**	0.694	−0.662	–	–	–
HNA-LS	–	**−0.515**	–	–	–	0.701	–	0.669	–	–	–	–	–
LNA	−0.796	–	0.782	−0.691	−0.718	–	–	–	−0.682	0.688	–	–	–
Viral abundance	−0.804	–	0.833	−0.680	−0.840	–	0.549	–	**−0.504**	0.923	–	–	–
PHP	−0.869	–	0.869	−0.789	−0.822	–	–	–	−0.676	–	–	–	–
VPR	0.724	–	−0.703	0.787	0.560	**−0.510**	–	**−0.521**	–	−0.676	–	–	–
**EASTERN SECTION**													
Prok. abundance	−0.932	0.655	0.913	–	−0.873	–	0.519	–	–	0.933	**0.829**	**−0.607**	–
HNA-HS	0.696	–	−0.691	–	0.626	–	–	–	0.724	−0.623	−1.000	0.804	–
HNA-LS	–	–	−0.543	–	–	–	–	–	–	**−0.504**	–	–	–
LNA	−0.851	0.598	0.858	–	−0.792	–	–	–	–	0.813	–	**−0.636**	–
Viral abundance	−0.640	0.524	0.643	–	−0.574	–	–	–	–	0.693	–	–	–
VirHigh	−0.582	–	0.568	–	−0.561	–	0.619	–	–	0.673	–	–	–
VirMed	–	–	–	–	–	–	–	–	0.783	–	–	–	–
VirLow	–	–	–	–	–	–	–	–	−0.728	–	–	–	–
PHP	−0.896	0.634	0.887	–	−0.822	–	0.564	–	–	–	–	−0.744	–
VPR	–	–	–	–	–	–	–	–	–	–	−0.943	0.783	–
FIC	0.895	−0.867	−0.867	–	0.867	–	–	–	0.783	−0.744	**−0.886**	–	–
Lysogenic VP	−0.943	0.943	0.943	–	−0.943	–	**0.883**	–	−0.943	**0.829**	–	–	–

Only significant results are shown (−0.5 > *r*_*s*_ > 0.5). Numbers in bold are 0.01 = p = 0.05. Dashes indicate no significant correlation for the specific parameter. Abbreviations: FIC, frequency of infected cells; FLC, frequency of lysogenically infected cells induced by mitomycin C; HNA-HS, percentage of high nucleic acid prokaryotes counted with high scatter; HNA-LS, percentage of high nucleic acid prokaryotes counted with low scatter; LNA, percentage of low nucleic acid prokaryotes; PHP, prokaryotic heterotrophic production; VirHigh, percentage of high fluorescent viruses; VirLow, percentage of low fluorescent viruses; VirMed, percentage of medium fluorescent viruses; VP, viral production; VPR, virus-to-prokaryote ratio.

**Figure 2:**
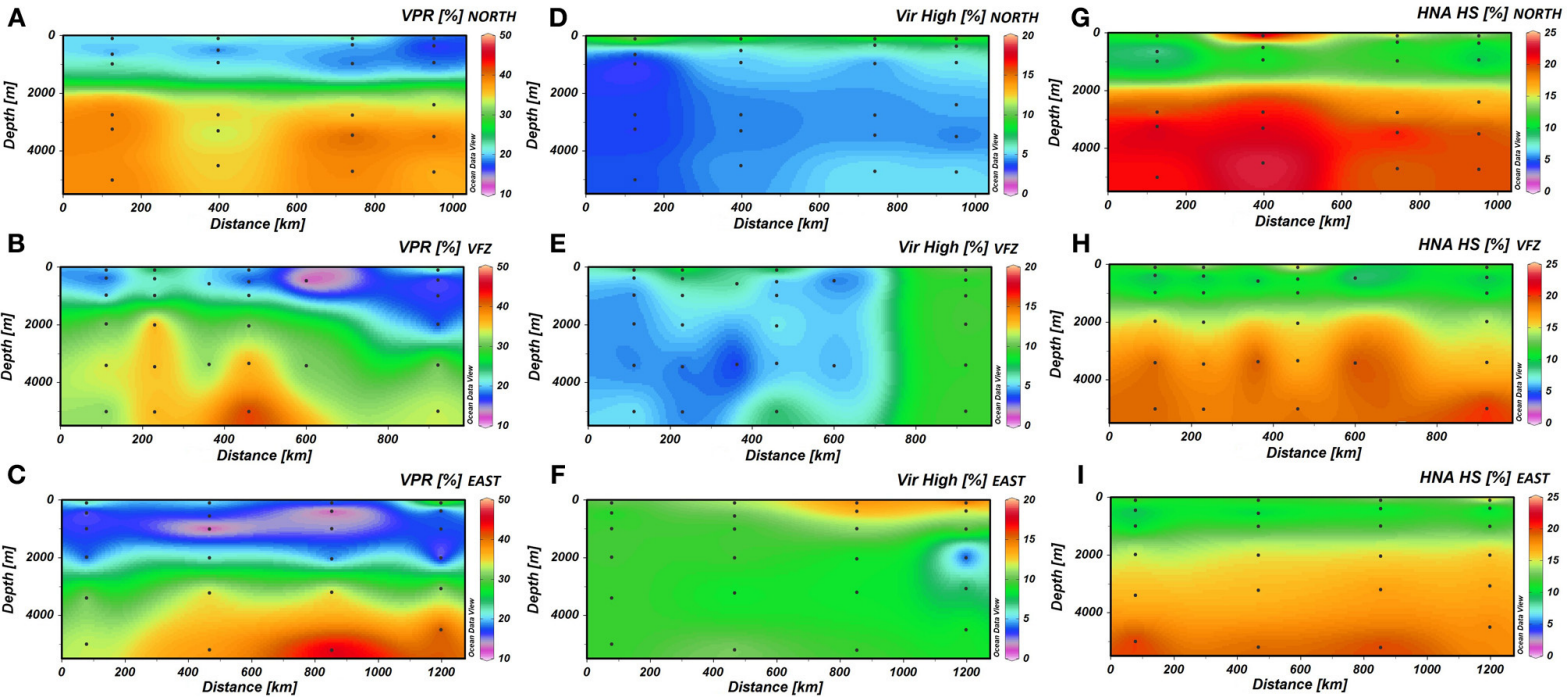
**The spatial distribution of the virus-to-prokaryote ratio (A–C) and the high fluorescence nucleic acid containing viral (D–F), and prokaryotic (G–I) cells north (upper panels), within (center panels) and east (lower panels) of the Vema Fracture Zone of the subtropical North Atlantic Ocean**. Each dot represents an individual sampling point. HNA HS, percentage of high nucleic acid containing prokaryotes counted with high-scatter; Vir High, percentage of high fluorescent viruses; VFZ, Vema Fracture Zone; VPR, Virus-to-Prokaryote Ratio.

The VPR generally increased with depth (Figures 2A–C) and was also significantly higher in the UNADW of the VFZ than of the eastern section (Table 1). Outside the VFZ, VPR was positively correlated to the FIC but not in the VFZ (Table 2).

In the VFZ, the percentage of the VirHigh fraction was significantly lower in the LNADW, AAIW, OMZ and 100 m depth (Figures 2D–F) than east of the VFZ. Table 1 depicts significant differences of the HNA-HS (Figures 2G–I), VirMed and VirLow populations in the VFZ compared to north (HNA-HS) and east (VirMed, VirLow) of the VFZ.

### Bacterial and archaeal community composition analyzed by T-RFLP

The number of OTUs obtained with the BacFwd and BacRev primer varied between 3 and 38 throughout the water column and did not exhibit depth-related trends (Table 3 and Table S4) nor consistent patterns between inside and outside the VFZ. Inside the VFZ, the number of OTUs obtained with both primers in the 100 m depth layer was significantly higher than in the deeper water masses. Also, the number of OTUs obtained with the BacRev primer in the LNADW was significantly lower in the northern than in the eastern section.

**Table 3:** **Number of OTUs of T-RFLP (forward primer) and RAPD-PCR data detected within the different water masses north, within and east of the Vema Fracture Zone in the (sub)tropical North Atlantic Ocean**.

	**BacFwd**			**ArchFwd**			**VirCRA**		**VirOPA**		
	**Avg (*SD*)**	**Range**	***N***	**Avg (*SD*)**	**Range**	***N***	**Avg (*SD*)**	**Range**	**Avg (*SD*)**	**Range**	***N***
**NORTHERN SECTION**											
100 m	19.50 (−)	9–30	2	6.75 (1.89)	4–8	4	9.75 (1.26)	8–11	12.25 (3.30)	10–17	4
OMZ	11.25 (10.21)	3–25	4	7.33 (2.52)	5–10	3	9.50 (1.91)	7–11	12.50 (1.29)	11–14	4
AAIW	7.67 (3.51)	4–11	3	6.00 (1.73)	5–8	3	8.00 (1.41)	6–9	**12.50** (2.38)	9–14	4
UNADW	15.67 (6.51)	9–22	3	7.00 (0.82)	6–8	4	10.00 (1.63)	8–12	12.50 (1.29)	11–14	4
LNADW	17.25 (6.24)	9–24	4	5.50 (1.29)	4–7	4	9.25 (2.06)	7–12	10.50 (5.07)	3–14	4
AABW	15.67 (8.33)	9–25	3	8.00 (3.00)	5–11	3	10.25 (1.71)	8–12	10.25 (1.71)	8–12	4
**VEMA FRACTURE ZONE**											
100 m	**22.67** (1.53)	21–24	3	8.00 (1.00)	7–9	3	9.33 (3.06)	6–10	8.67 (1.15)	8–10	3
OMZ	15.33 (1.15)	14–16	3	7.00 (−)	7	2	8.00 (5.20)	2–11	10.00 (2.00)	8–12	3
AAIW	**13.33** (0.58)	13–14	3	8.00 (2.83)	6–10	2	6.67 (3.21)	3–9	8.33 (2.08)	6–10	3
UNADW	**12.00** (−)	9–15	2	8.50 (−)	7–10	2	9.00 (5.20)	3–12	10.00 (3.00)	7–13	3
LNADW	**12.67** (3.79)	10–17	3	3.50 (−)	2–5	2	6.33 (3.79)	2–9	9.00 (3.46)	5–11	3
AABW	16.00 (3.00)	13–19	3	6.33 (1.15)	5–7	3	7.33 (2.08)	5–9	11.33 (4.73)	6–15	3
**EASTERN SECTION**											
100 m	22.25 (2.75)	19–15	4	5.75 (1.26)	4–7	4	14.50 (2.89)	11–18	12.75 (3.20)	8–15	4
OMZ	19.50 (1.73)	17–21	4	6.00 (1.41)	4–7	4	16.00 (2.16)	14–19	10.00 (4.08)	4–13	4
AAIW	16.50 (6.40)	10–22	4	6.25 (0.96)	5–7	4	11.00 (2.94)	7–14	**6.50** (3.11)	3–10	4
UNADW	19.50 (6.19)	11–25	4	5.75 (0.96)	5–7	4	12.00 (1.15)	11–13	10.50 (4.65)	3–15	4
LNADW	25.00 (5.06)	19–32	4	4.00 (1.41)	3–6	4	11.25 (3.30)	8–15	10.50 (5.20)	3–15	4
AABW	14.25 (5.50)	7–19	4	4.25 (0.96)	3–5	4	12.25 (2.87)	9–16	8.25 (3.10)	4–11	4

Abbreviations: Avg, average number of OTUs; SD, standard deviation; AABW, Antarctic Bottom Water; AAIW, Antarctic Intermediate Water; ArchFwd, archaeal forward primer; BacFwd, bacterial forward primer; LNADW, Lower North Atlantic Deep Water; N, number of samples; OMZ, oxygen minimum zone; OTU, operational taxonomic unit; UNADW, Upper North Atlantic Deep Water; VirCRA, viral primer CRA-22; VirOPA, viral primer OPA-13. Numbers in bold are significantly different (p ≤ 0.05).

**Figure 3:**
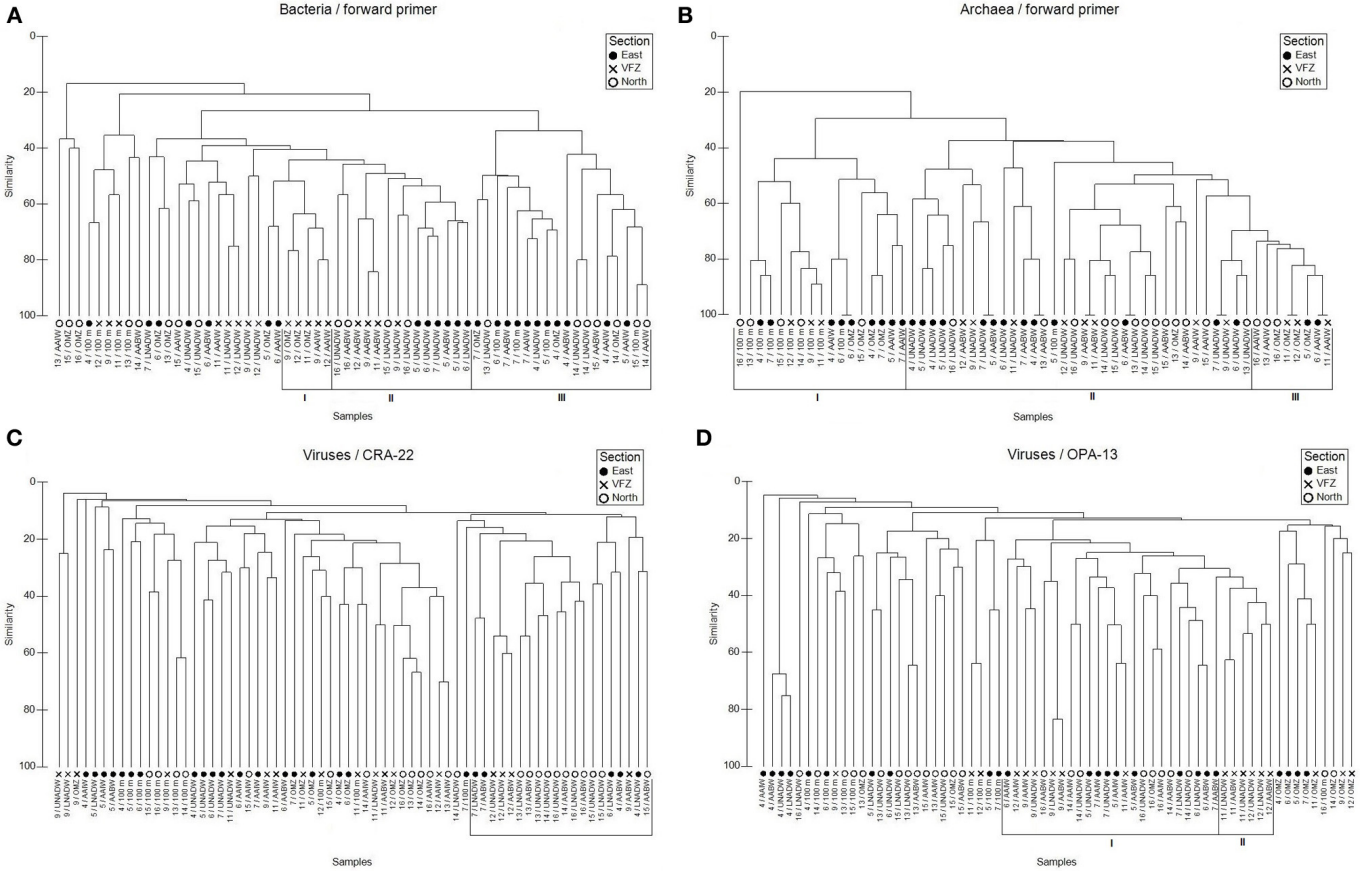
**Jaccard clustering analyses of bacterial (A), archaeal (B), and viral (C,D) communities sampled in different water masses north, within and east of the Vema Fracture Zone (VFZ), obtained by T-RFLP (using forward primers) and RAPD-PCR fingerprints**. Abbreviations: 100 m, bottom of euphotic layer; AABW, Antarctic Bottom Water; AAIW, Antarctic Intermediate Water; LNADW, Lower North Atlantic Deep Water; OMZ, Oxygen Minimum Zone; UNADW, Upper North Atlantic Deep Water; VFZ, Vema Fracture Zone. Latin numbers indicate different clusters.

Using the BacFwd primer, Jaccard analysis revealed one cluster of bacterial communities inhabiting the upper and lower NADW and the AABW of both the in- and outside of the VFZ (Figure 3A). A second cluster was dominated by bacterial communities of all sampled water masses outside the VFZ and another smaller cluster comprised bacterial communities of the OMZ and AAIW inside the VFZ (Figure 3A). Six major clusters were identified for the bacterial communities obtained with the BacRev primer (Figure S1A). Two clusters comprised bacterial communities of the eastern section (Figure S1A). Another two clusters contained bacterial communities of all water masses north of the VFZ and the biggest cluster was dominated by bacterial communities from the OMZ to the AABW inside the VFZ. Additionally, one cluster contained bacterial communities of the lower water masses (AAIW, upper and lower NADW, AABW) in- and outside the VFZ (Figure S1A).

The number of archaeal OTUs obtained with the ArchFwd and the ArchRev primer was rather homogenously distributed throughout the water column and ranged from 2 to 11 OTUs for the ArchFwd (Table 3) and from 1 to 7 OTUs for the ArchRev primer (Table S4). The archaeal communities obtained with the ArchFwd primer grouped into 3 clusters (Figure 3B). Two clusters comprised the archaeal communities of the upper water masses (100 m, OMZ and AAIW) in- and outside of the VFZ, one cluster was dominated by the deep-water masses (UNADW, LNADW, AABW) north, east and inside the VFZ with a similarity >40% (Figure 3B). Using the ArchRev primer for T-RFLP, only one archaeal community cluster was identifiable, dominated by communities of the deep-water masses (upper and lower NADW, AABW) north and east of the VFZ (Figure S1B).

Taken together, no clear clustering of the archaeal and bacterial communities of the deep-water masses between in- and outside the VFZ was apparent.

### Viral community composition analyzed by RAPD-PCR

Throughout the water column, the number of bands obtained with the VirOPA primer was significantly higher (Spearman's rank: *r*_*s*_ = 0.886, *p* = 0.019) than the number of bands obtained with the VirCRA primer, ranging from 3 to 17 and from 2 to 19, respectively (Table 3). Outside the VFZ, the number of VirOPA bands in the AAIW of the northern section was significantly higher than in the AAIW of the eastern section. However, the distribution of ubiquitous and unique bands did not show a clear trend neither with depth nor between inside and outside the VFZ. Cluster analysis using the VirCRA primer identified one pronounced cluster composed of deep-water viral communities in- and outside the VFZ (Figure 3C). Generally, the viral communities of the same water masses grouped into several small clusters. A similar pattern was found for the viral communities obtained with the VirOPA primer (Figure 3D), with a large cluster of lower water masses (AAIW, UNADW, LNADW, and AABW) in- and outside the VFZ and with one cluster of deep-water (UNADW, LNADW, AABW) viral communities inside the VFZ.

Collectively, a depth-related clustering of the viral community was evident with clusters of viral communities originating from the upper water masses and more pronounced clusters of the deep-water masses inside and outside the VFZ.

### Changes in prokaryotic and viral community composition with depth

**Figure 4:**
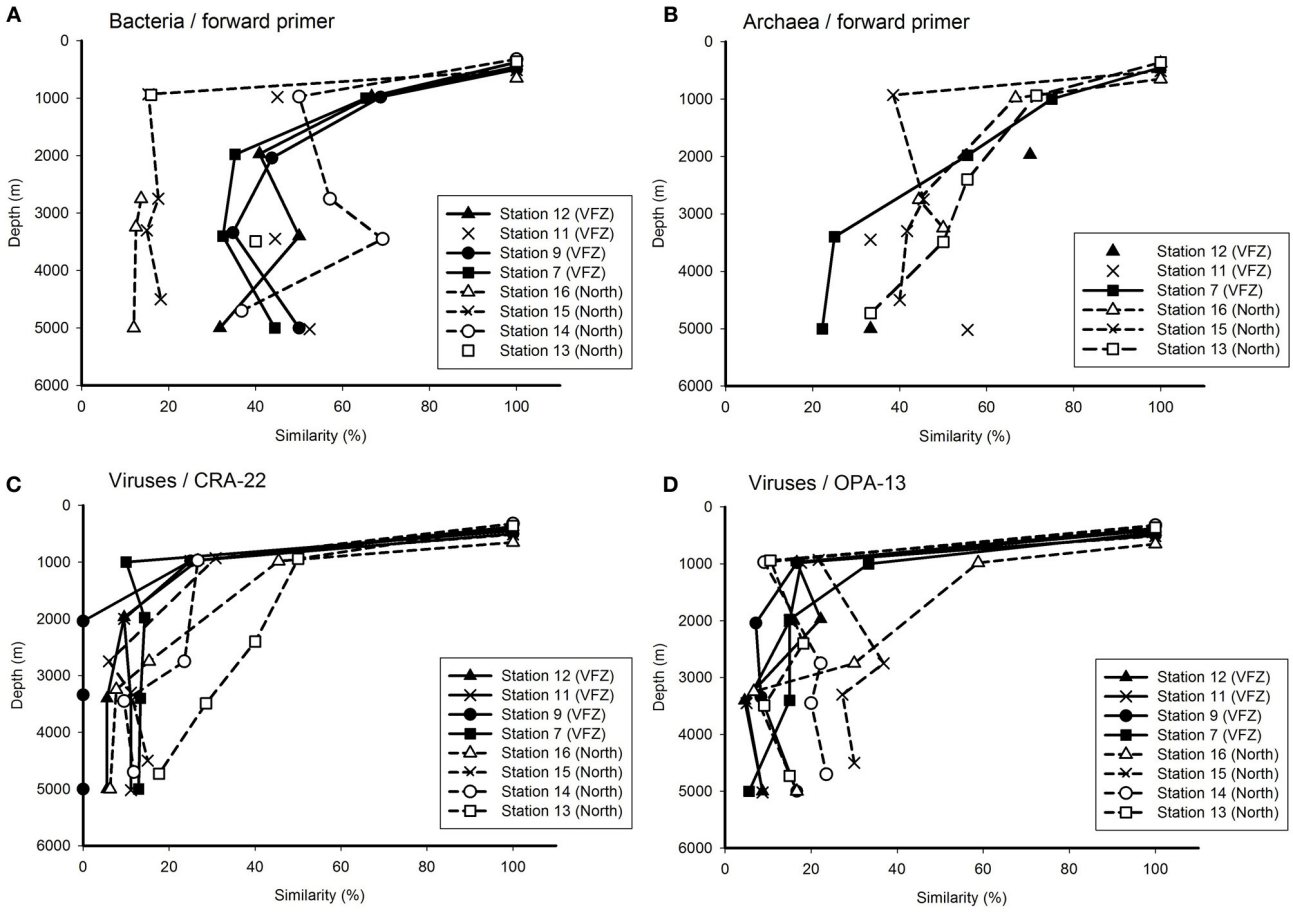
**Relationship between depth and Jaccard similarity of the bacterial (A), archaeal (B), and viral (C,D) community north and inside the Vema Fracture Zone (VFZ) in the (sub)tropical North Atlantic Ocean**. Each symbol represents the similarity of the community of a particular water mass compared to that of the oxygen minimum zone (approximately 500 m depth).

To obtain a more detailed insight into the changes in the bacterial, archaeal and viral community with depth inside the VFZ as compared to the northern section, we used the OMZ at approximately 500 m depth as reference. Generally, the similarity of the bacterial community obtained with the BacFwd primer decreased with depth inside the VFZ from the OMZ to the LNADW (3400 m depth) to 30–50% and with the BacRev primer to 25–60% (Figure 4A and Figure S2A). However, the northern stations did not show a clear trend with depth. The archaeal communities were generally characterized by a clear decrease in similarity with depth in the northern section and inside the VFZ. In contrast to the prokaryotic communities, the viral community obtained with both the CRA-22 and the OPA-13 primer showed a rapid decrease in similarity with depth from the OMZ to the UNADW and did not vary greatly below 2000 m inside the VFZ (0–18 %) (Figures 4C,D). At the northern section, however, the decrease in similarity of the viral community with depth was less pronounced than in the VFZ.

Taken together, our results indicate that the viral community was more intensely modified with depth in the deep-water masses inside the VFZ as compared to the northern section and as compared to the prokaryotic community.

### Variations of the microbial community composition with distance

**Figure 5:**
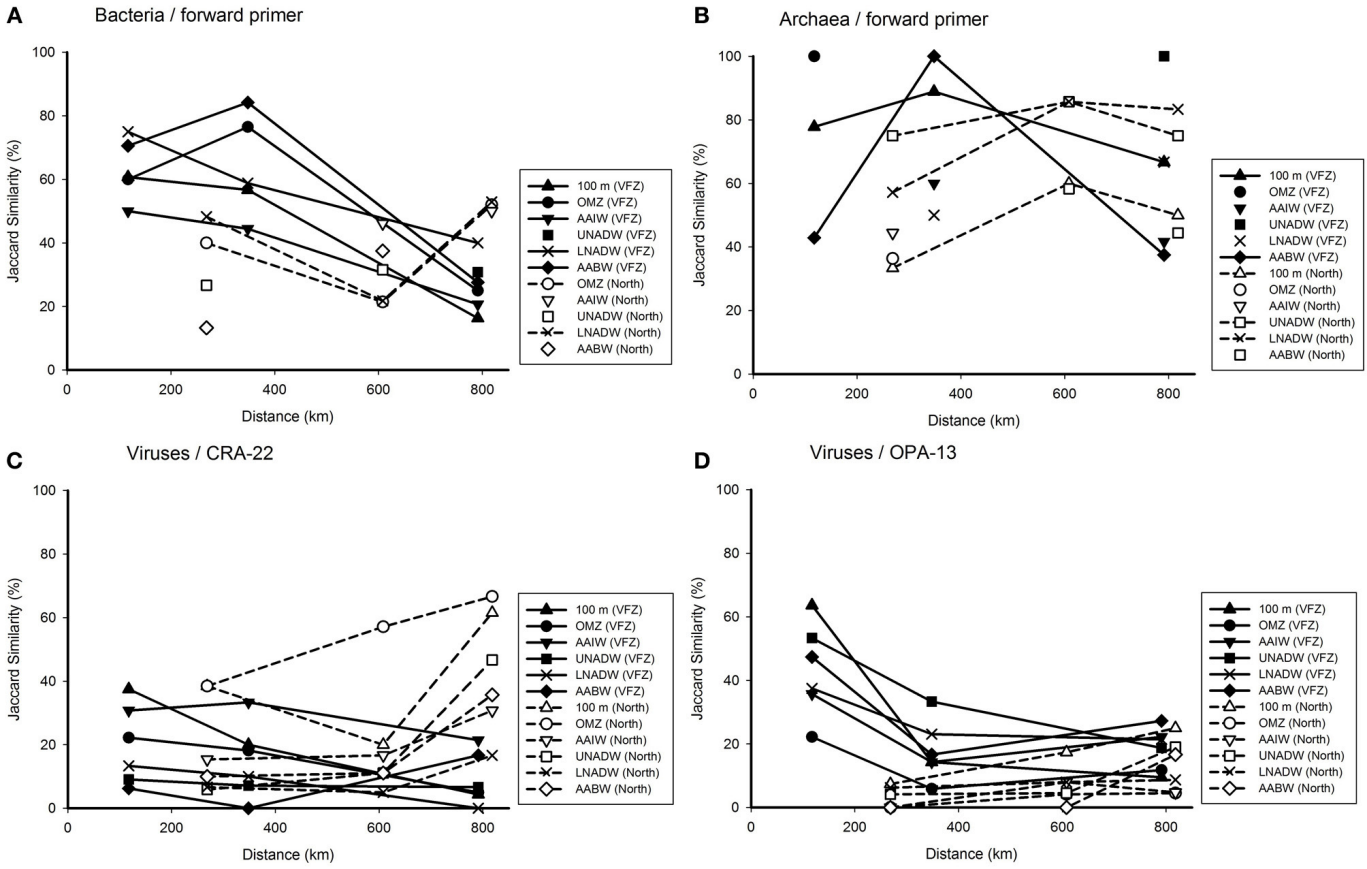
**Jaccard similarity plotted against the covered distance inside the VFZ as compared to the northern section, depicting alterations in the bacterial (A), archaeal (B), and viral (C,D) communities**. Each symbol represents the similarity of the community in the corresponding water mass to the previous station.

The effect of the geographic distance on the microbial community composition was tested for specific water masses along their flow through the VFZ (Figure 5). The similarity of the BCC generally decreased in all water masses sampled along their flow through the VFZ as compared to the northern section using the Bac Fwd primer (Figure 5A). A similar tendency was found with the BacRev primer (Figure S3A).

The few data obtained for the archaeal communities (Figures 5B and Figure S3B) did not reveal differences in ACC in the deep-water masses between the VFZ and the northern section.

The viral community of the deep-water masses (UNADW, LNADW, AABW) obtained with the VirCRA primer did not vary significantly with distance in the deep-water masses inside the VFZ and along the northern section (Figure 5C). The changes in the viral community over distance obtained with the VirOPA primer (Figure 5D) followed a different pattern with an overall higher similarity at the entrance of the VFZ than that obtained with the VirCRA primer. Generally the viral community obtained with the VirOPA primer was more stable in the deep-water masses (UNADW, LNADW, AABW) of the VFZ than along the northern section.

Taken together, inside the VFZ the similarity of the bacterial (and partly also the archaeal) community remained higher over distance than along the northern section. The viral community changed more rapidly than the bacterial community with distance in both the VFZ and along the northern section.

### Prokaryotic and viral production

**Figure 6:**
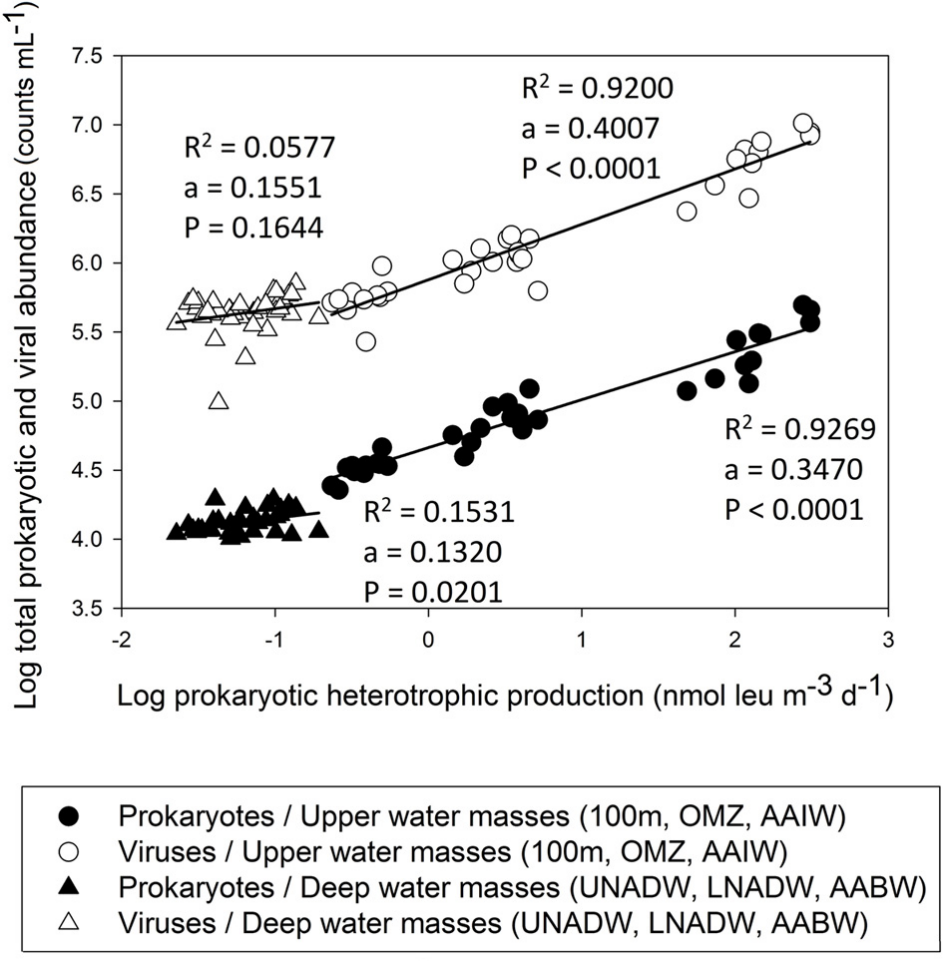
**Total prokaryotic and viral abundance versus prokaryotic heterotrophic production throughout the water column in the (sub)tropical North Atlantic Ocean**.

In the VFZ, PHP was significantly lower in the 100 m depth layer but significantly higher in the AAIW than in the northern section (Table 1). Pronounced differences in the slope between PHP and prokaryotic and viral abundance for the upper and mesopelagic (100 m, OMZ, AAIW) waters, on the one hand, and for the bathy- and abyssopelagic (UNADW, LNADW, AABW) water masses, on the other hand, were found (Figure 6).

Lytic VP significantly decreased with depth in the VFZ as well as outside (Table 4). The lytic VP in the LNADW was significantly higher in the VFZ compared to the eastern section. No significant trend between lytic VP and biological or physico-chemical parameters was discernable. Lysogenic VP showed no significant lateral or vertical trends but was positively correlated with prokaryotic abundance and depth east of the VFZ (Table 2).

**Table 4:** **Frequency of infected cells (FIC), frequency of lysogenized cells (FLC), lytic and viral production (VP) rates at different depth layers in the three sections**.

**Water mass**	**Depth (m)**	**FIC (%)**		**FLC (%)**		**Lytic VP (*N* × 10^3^ cells mL^−1^ h^−1^)**		**Lysogenic VP (*N* × 10^3^ cells mL^−1^ h^−1^)**	
		**Avg (*SD*)**	***N***	**Avg (*SD*)**	***N***	**Avg (*SD*)**	***N***	**Avg (*SD*)**	***N***
**NORTHERN SECTION**									
OMZ	459	21.2 (4.0)	4	2.9 (1.7)	3	42.1 (9.4)	4	8.6 (−)	1
LNADW	3371	32.8 (11.3)	4	12.2 (3.7)	3	12.3 (1.6)	4	3.9 (4.6)	3
AABW	4735	32.3 (18.3)	4	27.3 (19.8)	3	13.8 (10.9)	4	8.5 (8.2)	3
**VEMA FRACTURE ZONE**									
OMZ	428	21.7 (9.4)	3	9.2 (−)	2	41.3 (9.1)	3	9.7 (−)	2
LNADW	3398	25.9 (6.8)	3	11.9 (6.4)	3	**12.5** (1.5)	3	2.7 (1.4)	3
AABW	5010	31.6 (10.8)	3	10.1 (−)	2	13.6 (6.9)	3	4.3 (−)	2
**EASTERN SECTION**									
OMZ	442	23.1 (3.4)	4	13.0 (2.1)	3	39.7 (7.4)	4	30.1 (−)	2
LNADW	3220	39.7 (9.5)	4	22.5 (22.7)	3	**9.4** (0.6)	4	4.4 (−)	2
AABW	4974	61.5 (10.0)	4	19.3 (17.7)	3	10.8 (2.5)	4	1.4 (−)	2

Abbreviations: AABW, Antarctic Bottom Water; FIC, frequency of infected cells in % of total prokaryotic abundance; FLC, frequency of lysogenic cells; LNADW, Lower North Atlantic Deep Water; N, number of samples; OMZ, oxygen minimum zone; UNADW, Upper North Atlantic Deep Water; VP, viral production. Depth is given as average values. Numbers in bold indicate significant differences (p ≤ 0.05).

The FIC did not exhibit trends with depth in the VFZ while it increased in the eastern section from the OMZ to the AABW by approximately 50% (Table 4). No correlations of the FIC with biological or physico-chemical parameters were found in the VFZ but with HNA-LS and VPR in the northern section. Correlations of FIC with total prokaryotic abundance and the LNA population and the HNA-HS fraction, PHP as well as with salinity, potential temperature, depth and SiO_4_ were detected in the eastern section (Table 2).

Also, the FLC showed no significant trends in the VFZ but correlated with the HNA-HS, LNA, and VirMed populations in the northern section as well as with physico-chemical parameters (depth, oxygen and SiO_4_, salinity and potential temperature) (Table 2).

## Discussion

### Oceanographic conditions

As expected, the environmental variables measured (salinity, potential temperature, oxygen, PO_4_, SiO_4_, NO_3_) in the deep-water masses flowing through the VFZ were significantly modified (Tables S1, S2). Particularly, the changes in the physical and chemical characteristics of the UNADW and LNADW were obvious indicating that these water masses are influenced by mixing with the overlying AAIW and the underlying AABW, respectively, when funneled through the VFZ. Previous studies of the VFZ (Heezen et al., 1964; McCartney et al., 1991) report the presence of two sills, one on both ends of the VFZ, similar to the bottom topography of the more southern Romanche and Chain Fracture Zone (Fischer et al., 1996; Polzin et al., 1997; Ferron et al., 1998). These authors identified the sill as a source of turbulence in the AABW and pointed out that intense mixing could extend to 500 m above bottom downstream being responsible for strong modifications of the bottom water masses. However, also adjacent water masses well above the bottom are subjected to intense mixing at their margins due to the funneling of the water masses through the VFZ (McCartney et al., 1991). Furthermore, hydrothermal activity in the southern part of the VFZ has been reported (e.g., Auzende et al., 1990; Mamaloukas-Frangoulis et al., 1991) which might influence microbial diversity in the deep-water masses inside the VFZ distributing chemical compounds and microbial populations from the hydrothermal plume over large distances (Dick et al., 2013).

### Prokaryotic communities inside the VFZ

The decrease of total prokaryotic abundance by one order of magnitude from the euphotic to the abyssopelagic layer as observed in this study (Table 1) represents a typical pattern of the depth distribution of prokaryotes in the global oceanic water column (Aristegui et al., 2009). We could distinguish two prokaryotic populations according to the nucleic acid fluorescence intensity: high nucleic acid (HNA) content cells with two subunits based on the side scan scatters (low scatter vs. high scatter) and low nucleic acid (LNA) containing cells. The decreasing LNA-to-total HNA ratio of prokaryotes with depth is similar to that described for the Arctic and the Mediterranean Sea (Payet and Suttle, 2008; Winter et al., 2009). Also, Parada et al. (2007) reported an increasing fraction of HNA cells with depth in the Atlantic as depicted in Figures 2G–I. This has been interpreted as an indication that deep-sea prokaryotes harbor a larger genome than prokaryotes inhabiting the euphotic layer (Aristegui et al., 2009). A large genome in prokaryotes has been interpreted as an indication for an opportunistic lifestyle (Lauro et al., 2007). LNA cells have been thought to be less active than HNA cells, however, conflicting results on the relation between nucleic acid content and activity have been presented (Gasol et al., 1999; Lebaron et al., 2001; Zubkov et al., 2001, 2002; Servais et al., 2003; Longnecker et al., 2005; Mary et al., 2006).

### Changes in prokaryotic and viral communities with depth and distance

As shown for the Equatorial Pacific, the Mediterranean Sea and the English Channel, viruses with low fluorescence intensity (VirLow) are infecting primarily heterotrophic prokaryotes, whereas high fluorescence viruses (VirHigh) appear to be larger than VirLow and infect largely eukaryotic cells (Payet and Suttle, 2008; Evans et al., 2009; Winter et al., 2009). Inside the VFZ, the fractions of the three viral populations did not vary with depth. The VirMed and VirLow fractions dominated over the VirHigh fraction throughout the water column (Table 1). The higher contribution of the VirMed fraction in the UNADW of the VFZ than east of the VFZ might reflect changes in the dominant host between inside and outside of the VFZ. Also in a previous study of the subtropical Atlantic, the VirMed fraction was the most prominent fluorescence category of viruses (De Corte et al., 2010).

Generally, bacterial, archaeal and viral communities were depth stratified (Figure 4 and Figure S2) with distinct clusters for upper and deep-water masses (Figure 3, Figure S1). A similar depth stratification for this oceanic region has been reported previously (De Corte et al., 2010; Lekunberri et al., 2013) as well as for the Mediterranean Sea (De Corte et al., 2009; Yokokawa et al., 2010) and the North Pacific gyre (DeLong et al., 2006). This suggests, that physico-chemical factors such as temperature, hydrostatic pressure (Grossart and Gust, 2009) and organic matter quality such as sinking particles (Kiorboe et al., 2001; Moeseneder et al., 2001b) are the main factors controlling the prokaryotic and thus, indirectly also the viral community composition throughout the water column.

The successional changes in the prokaryotic and viral community composition within the VFZ over a distance of 790 km (i.e., Station 12–7), corresponding to a period of approximately 1 month (calculated from Demidov et al., 2007) of water mass transport through the channel revealed some differences in the development for prokaryotic and viral communities (Figure 5 and Figure S3). The prokaryotic communities retained a higher similarity inside the VFZ as compared to the viral communities, assuming that the latter is more dynamic in time than the prokaryotic community. However, we did not sample the exact same parcel of water as it moved through the VFZ but sampled the stations in the VFZ over a period of 1 week. Thus, the observed similarity patterns of the prokaryotic community might not directly reflect successional changes of the community caused by alterations of environmental factors in a given parcel of water but rather temporal dynamics of the communities in the different water masses (Hatosy et al., 2013 and references therein).

Also, we used fingerprinting methods to assess both, the prokaryotic and viral community composition. These fingerprinting techniques have a limited resolution. Hence, only the most abundant OTUs representing more than 0.5% of the community DNA are recovered by T-RFLP (Brown et al., 2005). Consequently, in the present study, any changes detected in the prokaryotic and viral community composition using these fingerprinting approaches are changes in the numerically dominant members of the respective community. Changes in less abundant (<0.5% of total DNA) members of the community remain unresolved by the applied methods.

### Virus-to-prokaryote ratio in the VFZ

The increase of the VPR with depth (Figures 2A–C) was caused by the lower decrease in viral abundance with depth as compared to prokaryotes as reported earlier (Weinbauer, 2004; Parada et al., 2007; Evans et al., 2009; De Corte et al., 2010, 2012). This increase in the VPR has been interpreted to be provoked by the lower decay rates of viruses in the deep sea in contrast to surface water viral communities and to a non-random distribution of prokaryotes and viruses in the deep sea (Parada et al., 2007). This assumption of a predominately non-random distribution of prokaryotes and viruses in the deep ocean is supported by the notion that prokaryotes inhabiting the deep sea generally harbor a larger genome and a higher content of genes indicative for an opportunistic, preferentially particle-attached life mode (DeLong et al., 2006; Parada et al., 2007; Herndl and Reinthaler, 2013).

### Prokaryotic heterotrophic (PHP) and viral production

Inside the VFZ, PHP was more variable than in the northern and eastern section of the transect (Table 1). In agreement with previous studies (e.g., De Corte et al., 2010, 2012; Evans and Brussaard, 2012), lytic VP significantly decreased with depth throughout the water column.

Lysogenic VP was variable throughout the water column and among the sections. Thus, lysogeny did not increase with depth as suggested by Weinbauer et al. (2003) who hypothesized that lysogeny is an adaption to low host abundance and activity and hence, lysogeny dominates over lysis as a prokaryotic mortality factor in the deep ocean. If VP in deep-waters would be predominantly lysogenic, total VP would be a function of induction events (Parada et al., 2007). The rate of induction depends on the environmental conditions (i.e., nutrient availability, presence of stressors, etc.) and thus, might not be effective to induce the lytic pathway in all prophages (Weinbauer and Suttle, 1999). In line of this, the reaction of prokaryotes to mitomycin C exposure depends on its concentration. Mitomycin C as a DNA mutagen might be toxic to some prokaryotes, especially in oligotrophic offshore environments (Jiang and Paul, 1996) but at the same time, not effective to induce all prophages (Ackermann and DuBow, 1987; Paul and Weinbauer, 2010; Thomas et al., 2011). Also, different prokaryotic strains may respond differently to the mitomycin C treatment (Jiang and Paul, 1996).

The FIC and FLC, i.e., the numbers of host cells that were lytically or lysogenically infected at the beginning of the incubation experiments, respectively, did not show any significant trends with depth neither inside nor outside the VFZ but FIC obviously exceeded FLC in all water masses (Table 4). If lysogeny would be the favored “life” strategy of phages in the deep ocean, one would expect a decrease of FIC concomitantly with an increase of FLC with depth as reported elsewhere (e.g., Weinbauer and Peduzzi, 1994; Weinbauer et al., 2003). Our FIC and FLC calculations presented in Table 4 are based on the difference in viral abundance between mitomycin C treated and untreated samples divided by the burst size (BS). For these calculations, the BS was assumed to be 30 based on data from open waters given in Parada et al. (2006). However, the actual BS at our study site might substantially deviate from 30. Another possibility for underestimating lysogeny might be that the incubation period was too short to efficiently induce the lytic cycle in prophages (Weinbauer et al., 2010). Given the rather long incubation period, however, it seems unlikely, that the latter is responsible for the low rates of lysogeny found in this study.

### Relationships between the microbial community and physico-chemical parameters

Both subpopulations of HNA cells, the HNA-LS and HNA-HS showed striking differences in their relationship to the physico-chemical parameters (Table 2). Inside the VFZ, the dominating HNA-LS group did not show any relation with depth and potential temperature but was positively correlated with PO_4_ and NO_3_ (Table 2). The HNA-HS group comprised a lower fraction in the OMZ, AAIW, and UNADW inside the VFZ than in the northern and eastern section. The negative relations with depth, total viral abundance, PHP and NO_3_ concentration distinguish the HNA-HS group from both the HNA-LS and LNA fractions (Table 2, Table S3). This supports the idea of Bouvier et al. (2007) to divide the prokaryotic fractions into different communities based on fluorescence and side scatter. Bouvier et al. (2007) found that HNA- and LNA-cells are positively and negatively correlated with PHP, respectively. However, in the VFZ, the LNA fraction was positively correlated with potential temperature and PHP indicating that the LNA group constitutes metabolically active cells rather than lysed or inactive cells as suggested previously (Gasol et al., 1999; Lebaron et al., 2002). The positive correlation of the VPR with depth (Table 2) obtained in this study has also been recently reported for other regions in the global ocean and seems to be a general feature (e.g., Evans et al., 2009; De Corte et al., 2010, 2012; Magiopoulos and Paraskevi, 2012).

## Conclusions

In summary, our study revealed that turbulent mixing of deep-water masses influences the activity of viruses and their prokaryotic hosts. Particularly the NADW and AABW are subjected to mixing during their transit through the VFZ, resulting in significant alterations of their physico-chemical parameters. In the VFZ, the UNADW and the LNADW sustained a significantly higher total viral abundance, VPR and lytic VP than outside the VFZ while the changes in the prokaryotic community composition were less pronounced in the VFZ than along the northern section. In contrast, the successional changes in the viral community of the deep waters were generally more pronounced than that of the prokaryotic community. Overall, it appears that mixing of deep-water masses funneled through fracture zones increases prokaryotic and viral activity but has only limited effects on the composition of the dominant members of the microbial and viral community.

## Author contributions

Christian Winter designed research, Simone Muck, Adam Klimiuk, Christian Winter, and Gerhard J. Herndl collected water at sea, and Simone Muck, Thomas Griessler, and Nicole Köstner analyzed the samples in the lab. Simone Muck and Gerhard J. Herndl wrote the paper.

### Conflict of interest statement

The authors declare that the research was conducted in the absence of any commercial or financial relationships that could be construed as a potential conflict of interest.

## Supplementary material

Figure S1 **Dendogram showing the Jaccard similarity of the bacterial (A) and archaeal (B) community from different water masses sampled north, within and east of the Vema Fracture Zone (VFZ) as revealed by T-RFLP, using reverse primers**. Latin numbers represent different clusters.

Figure S2 **Relationship between depth and Jaccard similarity of the bacterial (A) and archaeal (B) community north and inside the Vema Fracture Zone (VFZ) as determined by T-RFLP using reverse primers**. Each symbol represents the similarity in bacterial or archaeal community composition originating from a particular water mass in relation to the corresponding community of the oxygen minimum zone (approximately 500 m depth).

Figure S3 **Relation of the Jaccard similarity and covered distance of the water masses north and inside the Vema Fracture Zone, showing alterations in the bacterial (A) and archaeal (B) community composition obtained by T-RFLP (using the reverse primer)**. Symbols represent the similarities of the bacterial and archaeal communities to those in the corresponding water mass of the previously sampled station.

Table S1 **Physical characteristics of the main water masses sampled during the MOCA cruise divided into three sections (north, within and east of the Vema Fracture Zone) based on CTD profiles at the individual stations**. Numbers in bold without and with an asterisk are significantly different (*p* ≤ 0.05 and *p* ≤ 0.01, respectively). Only two values are available for oxygen concentrations within the eastern section. Abbreviations: 100 m, bottom of euphotic layer; AABW, Antarctic Bottom Water; AAIW, Antarctic Intermediate Water; OMZ, oxygen minimum zone; N, number of samples analyzed; LNADW, Lower North Atlantic Deep Water; Pot. Temp., potential temperature; UNADW, Upper North Atlantic Deep Water; VFZ, Vema Fracture Zone; Averages, standard deviation and range are indicated for all parameters.

Table S2 **Inorganic nutrient concentrations of the main water masses sampled during the MOCA cruise divided into three sections (north, within and east of the Vema Fracture Zone) based on CTD profiles at the individual stations**. Numbers in bold show significant differences (*p* ≤ 0.05). Abbreviations: 100 m, bottom of euphotic layer; AABW, Antarctic Bottom Water; AAIW, Antarctic Intermediate Water; OMZ, oxygen minimum zone; N, number of samples analyzed; LNADW, Lower North Atlantic Deep Water; UNADW, Upper North Atlantic Deep Water; Averages, standard deviation and range are indicated for all parameters.

Table S3 **Spearman's rank correlation coefficients determining the correlation between the prokaryotic and viral populations of the three sections**. Only statistically relevant (−0.5 > *r*_*s*_ > 0.5) data are shown. Numbers in bold are 0.01 ≤ *p* ≤ 0.05. Dashes indicate no significant correlation for the specific parameter. Abbreviations: HNA-HS, percentage of high nucleic acid prokaryotes counted with high scatter; HNA-LS, percentage of high nucleic acid prokaryotes counted with low scatter; LNA, percentage of low nucleic acid prokaryotes; VirHigh, percentage of high fluorescent viruses; VirLow, percentage of low fluorescent viruses; VirMed, percentage of medium fluorescent viruses.

Table S4 **Number of bacterial and archaeal operational taxonomic units (OTUs) revealed with reverse primers within the different water masses north, within and east of the Vema Fracture Zone in the (sub) tropical North Atlantic Ocean as revealed by T-RFLP**. Abbreviations: Avg, average number of OTUs; SD, standard deviation; AABW, Antarctic Bottom Water; AAIW, Antarctic Intermediate Water; ArchRev, archael reverse primer; BacRev, bacterial reverse primer; N, number of samples; OMZ, oxygen minimum zone; LNADW, Lower North Atlantic Deep Water; Range, minimal and maximal number of OTUs; OTU, operational taxonomic unit; UNADW, Upper North Atlantic Deep Water. Numbers in bold are significantly different (*p* ≤ 0.05).
