# Supplementary material for: Fracture zones in the Mid Atlantic Ridge lead to alterations in prokaryotic and viral parameters in deep-water masses
Source: Front Microbiol. 2014 Jun 2;5:264. doi: 10.3389/fmicb.2014.00264 (PMC4040922; doi:10.3389/fmicb.2014.00264)
Supplement: Table S1 — Physical characteristics of the main water masses sampled during the MOCA cruise divided into three sections (north, within and east of the Vema Fracture Zone) based on CTD profiles at the individual stations. Numbers in bold without and with an asterisk are significantly different (p ≤ 0.05 and p ≤ 0.01, respectively). Only two values are available for oxygen concentrations within the eastern section. Abbreviations: 100 m, bottom of euphotic layer; AABW, Antarctic Bottom Water; AAIW, Antarctic Intermediate Water; OMZ, oxygen minimum zone; N, number of samples analyzed; LNADW, Lower North Atlantic Deep Water; Pot. Temp., potential temperature; UNADW, Upper North Atlantic Deep Water; VFZ, Vema Fracture Zone; Averages, standard deviation and range are indicated for all parameters. [file DataSheet1.DOCX]

Table S1: Physical characteristics of the main water masses sampled during the MOCA cruise divided into three sections (north, within and east of the Vema Fracture Zone) based on CTD profiles at the individual stations. Numbers in bold without and with an asterisk are significantly different (p ≤ 0.05 and p ≤ 0.01, respectively). Only two values are available for oxygen concentrations within the eastern section.

Abbreviations: 100 m, bottom of euphotic layer; AABW, Antarctic Bottom Water; AAIW, Antarctic Intermediate Water; OMZ, oxygen minimum zone; N, number of samples analyzed; LNADW, Lower North Atlantic Deep Water; Pot. Temp., potential temperature; UNADW, Upper North Atlantic Deep Water; VFZ, Vema Fracture Zone; Averages, standard deviation and range are indicated for all parameters.

|  |  | Depth (m) | | Salinity | | | | Pot. Temp. (°C) | | | Oxygen (µmol kg^-1^) | | |  | |
| --- | --- | --- | --- | --- | --- | --- | --- | --- | --- | --- | --- | --- | --- | --- | --- |
|  | Water mass | Avg | Range | | Avg (SD) | Range | | | Avg (SD) | Range | | Avg (SD) | Range | N | |
| **Northern section** | 100 m | 100 | 98-100 | | **37.157** (0.341) | | 36.735-37.461 | | 22.713 (2.155) | 20.267-24.820 | | **176.92** (28.77) | 143.92-203.17 | | 4 |
|  | OMZ | 459 | 319-650 | | **35.235** (0.150) | | 35.013-35.345 | | 10.202 (0.610) | 9.624-10.885 | | 97.23 (10.04) | 88.78-111.79 | | 4 |
|  | AAIW | 953 | 934-975 | | 34.866 (0.062) | | 34.810-34.948 | | **5.823** (0.263) | 5.559-6.124 | | 130.84 (5.52) | 125.94-138.63 | | 4 |
|  | UNADW | 2661 | 2396-2752 | | **34.949*** (0.008) | | 34.940-34.957 | | 2.770 (0.164) | 2.643-3.009 | | 231.43 (3.23) | 227.28-235.08 | | 4 |
|  | LNADW | 3371 | 3246-3494 | | 34.917 (0.006) | | 34.912-34.925 | | 2.285 (0.062) | 2.231-2.356 | | 230.87 (2.32) | 228.05-233.59 | | 4 |
|  | AABW | 4735 | 4509-5003 | | **34.880** (0.003) | | 34.877-34.885 | | **1.884** (0.033) | 1.852-1.930 | | 231.57 (0.82) | 230.56-232.45 | | 4 |
| **Vema Fracture Zone** | 100 m | 100 | 99-101 | | **36.221** (0.043) | | 36.173-36.258 | | 18.381 (0.128) | 18.285-18.527 | | **113.14** (2.09) | 111.51-115.50 | | 3 |
|  | OMZ | 464 | 376-570 | | **34.903** (0.067) | | 34.812-34.968 | | 8.654 (0.728) | 7.659-9.416 | | 80.85 (5.73) | 75.66-89.90 | | 5 |
|  | AAIW | 975 | 969-981 | | 34.760 (0.008) | | 34.751-34.765 | | **5.200** (0.029) | 5.168-5.225 | | 133.68 (0.21) | 133.44-133.85 | | 3 |
|  | UNADW | 2002 | 1969-2038 | | **34.973*** (0.001) | | 34.971-34.973 | | 3.416 (0.015) | 3.402-3.432 | | **235.77** (1.32) | 234.74-237.25 | | 3 |
|  | LNADW | 3398 | 3340-3449 | | **34.911** (0.002) | | 34.910-34.914 | | **2.236** (0.021) | 2.218-2.261 | | 231.66 (1.23) | 230.79-233.84 | | 5 |
|  | AABW | 5010 | 5002-5023 | | **34.827** (0.001) | | 34.826-34.827 | | **1.361** (0.004) | 1.356-1.363 | | 228.77 (0.71) | 228.07-229.50 | | 3 |
| **Eastern section** | 100 m | 100 | 100-101 | | **36.210** (0.495) | | 35.477-36.556 | | 17.245 (2.382) | 13.794-19.180 | | 124.66 (11.83) | 116.29-133.03 | | 4 (2) |
|  | OMZ | 442 | 374-551 | | 35.185 (0.197) | | 34.980-35.373 | | 10.073 (1.295) | 8.554-11.324 | | 63.56 (12.36) | 49.49-79.54 | | 4 |
|  | AAIW | 998 | 995-1002 | | 34.871 (0.056) | | 34.820-34.940 | | 5.663 (0.257) | 5.407-5.946 | | 129.03 (1.51) | 127.09-130.49 | | 4 |
|  | UNADW | 2005 | 1976-2040 | | 34.977 (0.005) | | 34.972-34.983 | | 3.436 (0.034) | 3.398-3.476 | | **224.60** (5.36) | 218.60-229.58 | | 4 |
|  | LNADW | 3220 | 3070-3397 | | **34.924** (0.009) | | 34.914-34.935 | | **2.374** (0.083) | 2.276-2.466 | | 229.93 (2.11) | 227.48-232.39 | | 4 |
|  | AABW | 4974 | 4502-5198 | | 34.875 (0.011) | | 34.864-34.890 | | 1.812 (0.109) | 1.708-1.962 | | 231.61 (2.54) | 228.33-234.52 | | 4 |

Table S2: Inorganic nutrient concentrations of the main water masses sampled during the MOCA cruise divided into three sections (north, within and east of the Vema Fracture Zone) based on CTD profiles at the individual stations. Numbers in bold show significant differences (p ≤ 0.05). Abbreviations: 100 m, bottom of euphotic layer; AABW, Antarctic Bottom Water; AAIW, Antarctic Intermediate Water; OMZ, oxygen minimum zone; N, number of samples analyzed; LNADW, Lower North Atlantic Deep Water; UNADW, Upper North Atlantic Deep Water; Averages, standard deviation and range are indicated for all parameters.

|  |  | Depth (m) | | | PO_4_ (µM) | | SiO_4_ (µM) | | | | NO_3_ (µM) | | | |  |
| --- | --- | --- | --- | --- | --- | --- | --- | --- | --- | --- | --- | --- | --- | --- | --- |
|  | Water mass | Avg | Range | Avg (SD) | | Range | | Avg (SD) | Range | Avg (SD) | | Range | | N | |
| **Northern section** | 100 m | 100 | 98-100 | 0.156 (0.207) | | 0.009-0.449 | | **1.115** (0.652) | 0.567-1.947 | **2.288** (3.207) | | | 0.002-6.810 | 8 | |
|  | OMZ | 459 | 319-795 | **1.821** (0.122) | | 1.742-1.993 | | 13.619 (1.073) | 12.458-14.598 | **29.264** (1.549) | | | 28.007-31.520 | 8 | |
|  | AAIW | 953 | 934-1069 | 2.179 (0.116) | | 2.030-2.288 | | 24.991 (1.627) | 22.942-22.942 | 32.902 (1.600) | | | 30.759-34.294 | 8 | |
|  | UNADW | 2661 | 2396-2752 | **1.459** (0.042) | | 1.397-1.485 | | **31.666** (4.114) | 25.497-33.875 | **21.806** (0.604) | | | 20.902-22.155 | 8 | |
|  | LNADW | 3371 | 3246-3499 | 1.529 (0.009) | | 1.521-1.539 | | 40.893 (0.874) | 40.016-41.989 | 22.645 (0.118) | | | 22.481-22.741 | 8 | |
|  | AABW | 4735 | 4509-5004 | **1.593** (0.012) | | 1.579-1.606 | | **50.153** (0.775) | 49.082-50.916 | **23.485** (0.128) | | | 23.319-23.613 | 8 | |
| **Vema Fracture Zone** | 100 m | 100 | 99-100 | 0.813 (0.043) | | 0.764-0.843 | | **4.449** (0.310) | 4.092-4.652 | **13.910** (0.747) | | | 13.059-14.454 | 3 | |
|  | OMZ | 464 | 376-570 | **2.200** (0.091) | | 2.095-2.325 | | 17.888 (1.960) | 15.841-20.567 | **34.972** (0.903) | | | 33.990-36.023 | 5 | |
|  | AAIW | 975 | 969-981 | 2.273 (0.007) | | 2.266-2.281 | | 27.787 (0.162) | 27.691-27.974 | 33.977 (0.038) | | | 33.932-34.001 | 3 | |
|  | UNADW | 2002 | 1969-2038 | **1.346** (0.019) | | 1.324-1.359 | | **20.285** (0.985) | 19.150-20.916 | **20.381** (0.255) | | | 20.093-20.578 | 3 | |
|  | LNADW | 3398 | 3340-3449 | 1.510 (0.017) | | 1.485-1.532 | | **41.071** (0.978) | 39.904-42.429 | 22.425 (0.219) | | | 22.059-22.639 | 5 | |
|  | AABW | 5010 | 5002-5023 | **1.741** (0.004) | | 1.738-1.745 | | **67.487** (0.042) | 67.442-67.525 | **25.457** (0.019) | | | 25.440-25.477 | 3 | |
| **Eastern section** | 100 m | 100 | 100-101 | 1.001 (0.438) | | 0.027-1.655 | | 4.513 (2.578) | 3.066-8.365 | 16.259 (7.127) | | | 11.986-26.886 | 7 | |
|  | OMZ | 442 | 374-597 | 2.067 (0.198) | | 0.871-2.252 | | 15.169 (2.908) | 12.272-18.424 | 33.757 (2.647) | | | 31.018-36.724 | 7 | |
|  | AAIW | 998 | 772-1037 | 2.211 (0.063) | | 1.701-2.267 | | 25.545 (1.167) | 24.143-26.710 | 33.170 (0.661) | | | 32.292-33.784 | 7 | |
|  | UNADW | 2005 | 1040-2081 | **1.467** (0.046) | | 1.419-1.752 | | 23.442 (1.758) | 21.698-25.387 | **21.991** (0.725) | | | 21.249-22.744 | 7 | |
|  | LNADW | 3220 | 2969-3397 | 1.513 (0.008) | | 1.505-1.563 | | **38.740** (0.534) | 38.367-39.531 | 22.411 (0.223) | | | 22.102-22.632 | 7 | |
|  | AABW | 4974 | 3902-5198 | 1.607 (0.009) | | 1.590-1.617 | | 52.169 (2.129) | 49.398-54.590 | 23.778 (0.042) | | | 23.736-23.834 | 7 | |

Table S3: Spearman’s rank correlation coefficients determining the correlation between the prokaryotic and viral populations of the three sections. Only statistically relevant (-0.5 > r_s_ > 0.5) data are shown. Numbers in bold are 0.01 ≤ p ≤ 0.05. Dashes indicate no significant correlation for the specific parameter. Abbreviations: HNA-HS, percentage of high nucleic acid prokaryotes counted with high scatter; HNA-LS, percentage of high nucleic acid prokaryotes counted with low scatter; LNA, percentage of low nucleic acid prokaryotes; VirHigh, percentage of high fluorescent viruses; VirLow, percentage of low fluorescent viruses; VirMed, percentage of medium fluorescent viruses.

| **Northern section** | Prok. abund. | HNA-HS | HNA-LS | LNA | Viral abund. | VirLow | VirMed | VirHigh |
| --- | --- | --- | --- | --- | --- | --- | --- | --- |
| Prok. abundance | - | -0.581 | - | 0.523 | 0.962 | -0.771 | 0.620 | 0.689 |
| HNA-HS | 0.523 | - | -0.606 | -0.672 | -0.586 | 0.578 | -0.743 | - |
| HNA-LS | - | -0.606 | - | - | - | - | 0.513 | - |
| LNA | 0.523 | -0.672 | - | - | 0.528 | - | - | - |
| Viral abundance | 0.962 | -0.586 | - | 0.528 | - | -0.716 | 0.576 | 0.672 |
| VirLow | -0.771 | 0.578 | - | - | -0.716 | - | -0.918 | -0.724 |
| VirMed | 0.620 | **0.513** | **0.513** | - | 0.576 | -0.918 | - | - |
| VirHigh | 0.689 | - | - | - | 0.672 | -0.724 | - | - |
| **Vema Fracture Zone** |  |  |  |  |  |  |  |  |
| Prok. abundance | - | -0.670 | - | 0.688 | 0.935 | - | - | - |
| HNA-HS | -0.670 | - | -0.565 | -0.875 | -0.606 | - | - | - |
| HNA-LS | - | -0.565 | - | - | - | - | - | - |
| LNA | 0.688 | -0.875 | - | - | 0.596 | - | - | - |
| Viral abundance | 0.935 | -0.606 | - | 0.596 | - | **-0.514** | - | 0.548 |
| VirLow | - | - | - | - | **-0.514** | - | -0.699 | -0.607 |
| VirMed | - | - | - | - | - | -0.966 | - | - |
| VirHigh | - | - | - | - | 0.548 | -0.607 | - | - |
| **Eastern section** |  |  |  |  |  |  |  |  |
| Prok. abundance | - | -0.677 | -0.528 | 0.851 | 0.683 | - | - | 0.652 |
| HNA-HS | -0.677 | - | - | -0.667 | - | - | - | - |
| HNA-LS | -0.528 | - | - | -0.746 | - | - | - | - |
| LNA | 0.851 | -0.667 | -0.746 | - | 0.559 | - | - | **0.512** |
| Viral abundance | 0.683 | - | - | 0.559 | - | - | - | 0.628 |
| VirLow | - | - | - | - | - | - | -0.959 | - |
| VirMed | - | - | - | - | - | -0.959 | - | - |
| VirHigh | 0.652 | - | - | **0.512** | 0.628 | - | - | - |

Table S4: Number of bacterial and archaeal operational taxonomic units (OTUs) revealed with reverse primers within the different water masses north, within and east of the Vema Fracture Zone in the (sub)tropical North Atlantic Ocean as revealed by T-RFLP.

Abbreviations: Avg, average number of OTUs; SD, standard deviation; AABW, Antarctic Bottom Water; AAIW, Antarctic Intermediate Water; ArchRev, archael reverse primer; BacRev, bacterial reverse primer; N, number of samples; OMZ, oxygen minimum zone; LNADW, Lower North Atlantic Deep Water; Range, minimal and maximal number of OTUs; OTU, operational taxonomic unit; UNADW, Upper North Atlantic Deep Water. Numbers in bold are significantly different (p ≤ 0.05).

|  |  | **BacRev** | |  |  | **ArchRev** | |  |
| --- | --- | --- | --- | --- | --- | --- | --- | --- |
|  |  | Avg (SD) | Range | N |  | Avg (SD) | Range | N |
| **Northern section** | 100 m | 25.00 ( - ) | 12-38 | 2 |  | 3.75 (1.89) | 1-5 | 4 |
|  | OMZ | 11.25 (7.93) | 6-23 | 4 |  | 4.33 (0.58) | 4-5 | 3 |
|  | AAIW | 4.00 (1.00) | 3-5 | 3 |  | 3.67 (0.58) | 3-4 | 3 |
|  | UNADW | 12.67 (7.37) | 7-21 | 3 |  | 6.00 (1.15) | 5-7 | 4 |
|  | LNADW | **12.00** (5.10) | 7-19 | 4 |  | 5.50 (1.29) | 4-7 | 4 |
|  | AABW | 14.33 (6.81) | 9-22 | 3 |  | 6.00 (1.73) | 4-7 | 3 |
| **Vema Fracture Zone** | 100 m | **21.67** (4.73) | 18-27 | 3 |  | 6.00 (1.00) | 5-7 | 3 |
|  | OMZ | 14.67 (0.58) | 14-15 | 3 |  | 4.00 ( - ) | 4 | 2 |
|  | AAIW | 14.00 (3.46) | 10-16 | 3 |  | 6.00 ( - ) | 5-7 | 2 |
|  | UNADW | **9.00** ( - ) | 8-10 | 2 |  | 4.50 ( - ) | 4-5 | 2 |
|  | LNADW | **9.00** (2.00) | 7-11 | 3 |  | 3.00 ( - ) | 2-4 | 2 |
|  | AABW | **11.67** (1.15) | 11-13 | 3 |  | 5.00 (1.00) | 4-6 | 3 |
| **Eastern section** | 100 m | 20.25 (4.57) | 17-27 | 4 |  | 3.75 (0.50) | 3-4 | 4 |
|  | OMZ | 18.00 (4.55) | 13-24 | 4 |  | 3.50 (1.29) | 2-5 | 4 |
|  | AAIW | 14.75 (8.77) | 6-26 | 4 |  | 4.75 (0.50) | 4-5 | 4 |
|  | UNADW | 19.25 (9.18) | 7-29 | 4 |  | 3.75 (1.50) | 3-6 | 4 |
|  | LNADW | **23.50** (5.07) | 19-30 | 4 |  | 3.75 (2.22) | 2-7 | 4 |
|  | AABW | 12.50 (5.26) | 8-20 | 4 |  | 4.00 (1.41) | 3-6 | 4 |
