# Supplementary material for: Fracture zones in the Mid Atlantic Ridge lead to alterations in prokaryotic and viral parameters in deep-water masses
Source: Front Microbiol. 2014 Jun 2;5:264. doi: 10.3389/fmicb.2014.00264 (PMC4040922; doi:10.3389/fmicb.2014.00264)
Supplement: Figure S1 — Dendogram showing the Jaccard similarity of the bacterial (A) and archaeal (B) community from different water masses sampled north, within and east of the Vema Fracture Zone (VFZ) as revealed by T-RFLP, using reverse primers. Latin numbers represent different clusters. [file 63841_Muck_Data_Sheet_3.PDF › 63841_Muck_Data_Sheet_3.PDF]

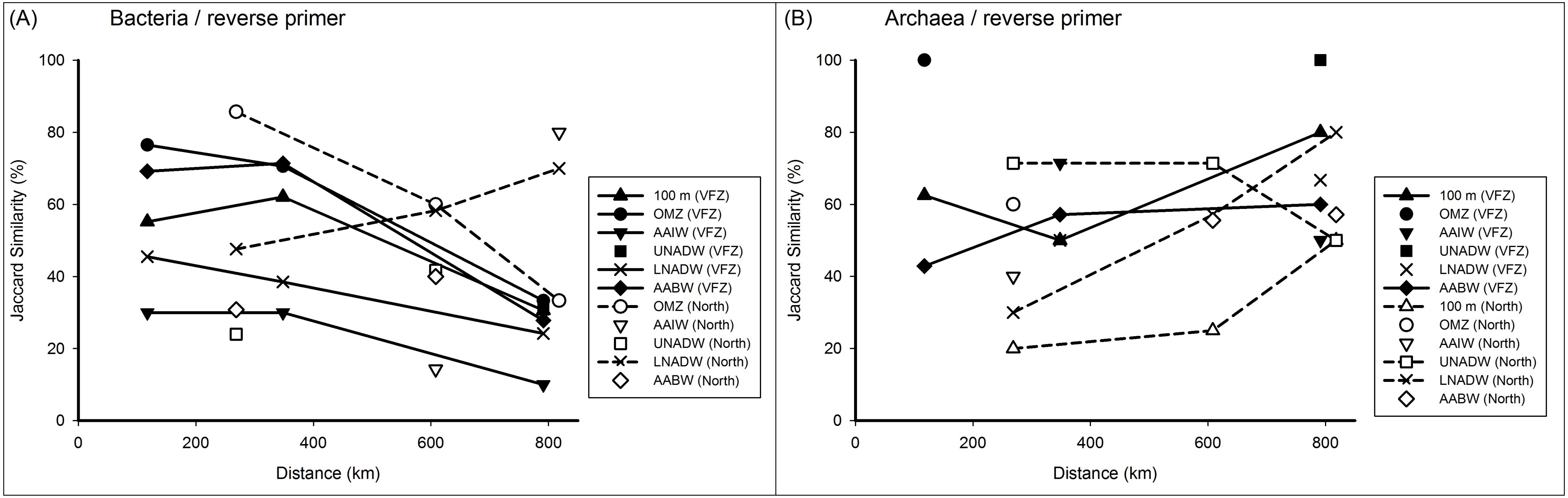
(A)
Bacteria / reverse primer
Jaccard Similarity (%)
0
20
40
60
80
100
0
200
400
600
800
Distance (km)
100 m (VFZ)
OMZ (VFZ)
AAIW (VFZ)
UNADW (VFZ)
LNADW (VFZ)
AABW (VFZ)
OMZ (North)
AAIW (North)
UNADW (North)
LNADW (North)
AABW (North)
(B)
Archaea / reverse primer
Jaccard Similarity (%)
0
20
40
60
80
100
0
200
400
600
800
Distance (km)
100 m (VFZ)
OMZ (VFZ)
AAIW (VFZ)
UNADW (VFZ)
LNADW (VFZ)
AABW (VFZ)
100 m (North)
OMZ (North)
AAIW (North)
UNADW (North)
LNADW (North)
AABW (North)
